# Glycolytic reprogramming in ovarian cancer: mechanisms, immune crosstalk, and therapeutic implications

**DOI:** 10.3389/fimmu.2026.1850399

**Published:** 2026-06-03

**Authors:** Jiao Fu, Yi Dai, Qiaoling Wang, Xiayu Qian

**Affiliations:** 1 Department of Obstetrics and Gynecology, Affiliated Hospital of Nantong University, School of Medicine, Nantong University, Nantong, Jiangsu, China; 2 Department of Pulmonary and Critical Care Medicine, Shanghai Pulmonary Hospital, School of Medicine, Tongji University, Shanghai, China; 3 Maternal and Child Health Care and Family Planning Service Center, Nantong, Jiangsu, China

**Keywords:** chemoresistance, glycolysis, immune crosstalk, metabolic reprogramming, ovarian cancer

## Abstract

Ovarian cancer is characterized by extensive peritoneal dissemination, frequent recurrence, and chemoresistance. Glycolytic reprogramming has emerged as a central metabolic adaptation in ovarian cancer, but its significance extends beyond increased glucose consumption. In this review, we summarize how key glycolytic regulators, including GLUT1, HK2, PFKFB3, PDK1, and LDHA, are controlled by oncogenic, microenvironmental, and non-coding RNA-mediated pathways to reshape tumor metabolism. We emphasize that glycolysis supports ovarian cancer progression by promoting biosynthetic activity, redox balance, invasive dissemination, stem-like plasticity, and therapy resistance. Importantly, this review highlights glycolysis as an immunometabolic regulator of the ovarian tumor microenvironment. Lactate accumulation, macrophage reprogramming, IL-1β/NF-κB signaling, PD-L1 induction, and CD4+ T-cell metabolic remodeling collectively contribute to immune escape. Targeting glycolytic pathways may therefore provide therapeutic opportunities not only to suppress tumor growth but also to enhance chemotherapy and immunotherapy. However, metabolic heterogeneity, compensatory pathway activation, limited biomarkers, and insufficient clinical validation remain major challenges. A glycolysis-centered understanding of ovarian cancer may support biomarker-guided combination strategies and improve translational therapeutic design.

## Highlights

- Glycolytic reprogramming is a central metabolic feature of ovarian cancer and is driven by key regulators such as GLUT1, HK2, PFKFB3, PDK1, and LDHA.
- Enhanced glycolysis promotes ovarian cancer progression by supporting proliferation, metastasis, stemness, chemoresistance, and metabolic plasticity.
- Glycolysis also contributes to immune escape through lactate-dependent macrophage remodeling, PD-L1-associated immunosuppression, and T-cell metabolic dysfunction.

## 1 Introduction

Ovarian cancer remains one of the most lethal gynecologic malignancies worldwide (1–3). Its high mortality is largely attributable to insidious early symptoms, frequent diagnosis at an advanced stage, extensive intraperitoneal dissemination, and the near-inevitable development of recurrence after initial therapy. Although cytoreductive surgery combined with platinum-based chemotherapy, followed in selected patients by targeted maintenance strategies such as PARP inhibition or anti-angiogenic treatment, has improved disease management, durable control is still limited in many cases (4–8).

Metabolic reprogramming is now recognized as a fundamental hallmark of malignant progression. In ovarian cancer, this metabolic plasticity extends beyond a simple increase in nutrient consumption and reflects a coordinated adaptation that supports proliferation, survival under stress, metastatic dissemination, and treatment resistance (9–11). Among the metabolic pathways implicated in this process, glycolysis has attracted particular attention. Even in the presence of oxygen, ovarian cancer cells frequently exhibit a glycolytic phenotype characterized by increased glucose uptake, accelerated lactate production, and preferential routing of carbon intermediates toward biosynthesis and redox balance (12). This reprogramming is not merely a passive consequence of rapid growth; rather, it is actively enforced by oncogenic signaling networks and contributes directly to tumor fitness. Core glycolytic nodes such as GLUT1, HK2, LDHA, and PFKFB3 are frequently dysregulated in ovarian cancer (13).

Interest in glycolysis in ovarian cancer has also expanded because its consequences extend far beyond ATP generation. Enhanced glycolytic flux provides metabolic intermediates for nucleotide, amino acid, and lipid synthesis, while lactate accumulation acidifies the tumor microenvironment and reshapes intercellular communication (14–16). In parallel, glycolysis interfaces with major regulatory circuits such as PI3K/AKT/HIF-1α, MYC, Wnt/β-catenin, p53, and inflammatory signaling pathways, thereby linking nutrient use to cell-cycle progression, epithelial–mesenchymal plasticity, oxidative stress adaptation, and resistance to apoptosis.

Importantly, glycolytic reprogramming in ovarian cancer should not be viewed as a tumor cell-intrinsic phenomenon alone. Emerging evidence indicates that it also shapes the immune landscape of the tumor microenvironment. Lactate-rich ovarian tumors can promote immunosuppressive signaling, facilitate tumor-associated macrophage remodeling, and enhance PD-L1-associated immune escape (17, 18). In addition, ovarian cancer cells can influence the metabolic state of CD4+ T cells and favor an immunosuppressive phenotype, highlighting that glucose metabolism is intimately connected to immune dysfunction in this disease (19–21). These findings suggest that glycolysis lies at the intersection of tumor progression, therapeutic resistance, and immune evasion, making it an attractive framework for mechanistic synthesis and translational targeting.

In this review, we provide a comprehensive overview of glycolytic reprogramming in ovarian cancer, emphasizing its molecular drivers, roles in tumor progression and therapy resistance, and emerging immunometabolic crosstalk. Unlike previous reviews on cancer glycolysis or ovarian cancer metabolism, we particularly highlight the “glycolysis–lactate–immune suppression” axis as a central mechanism linking metabolic adaptation to chemoresistance and poor responsiveness to immunotherapy. In addition, we integrate ovarian cancer-specific features such as ascites, peritoneal dissemination, and a macrophage-enriched tumor microenvironment. We further discuss therapeutic opportunities for targeting glycolysis, both as monotherapy and in combination with chemotherapy or immunotherapy, and highlight key challenges and future directions for translating mechanistic insights into clinically actionable strategies, including biomarker-guided and combination approaches. By connecting molecular mechanisms, tumor progression, immune escape, and therapeutic strategies, this review provides a cohesive framework that links mechanistic understanding to translational and clinical relevance.

## 2 Overview of glycolytic reprogramming in ovarian cancer

### 2.1 The glycolytic pathway and the Warburg effect

Glycolysis is a highly conserved metabolic pathway in which glucose is sequentially converted into pyruvate through a series of cytosolic enzymatic reactions. The pathway begins with glucose uptake across the plasma membrane, a step commonly mediated by facilitative glucose transporters such as GLUT1, followed by phosphorylation by hexokinase (HK) to generate glucose-6-phosphate and thereby trap glucose within the cell (22–25). Subsequent reactions convert glucose-6-phosphate into fructose-6-phosphate and then into fructose-1, 6-bisphosphate, the latter step being catalyzed by phosphofructokinase-1 (PFK1) and functionally reinforced by PFKFB3, which produces fructose-2, 6-bisphosphate, a potent allosteric activator of PFK1 (26). Downstream, phosphoenolpyruvate is converted to pyruvate by pyruvate kinase (PK), and pyruvate is either transported into mitochondria for oxidative metabolism or reduced to lactate by lactate dehydrogenase (LDH), particularly LDHA, with concomitant regeneration of NAD+, which sustains continued glycolytic flux.

Under normal oxygenated conditions, differentiated cells preferentially oxidize pyruvate in mitochondria through the tricarboxylic acid cycle and oxidative phosphorylation because this route generates ATP more efficiently than glycolysis alone. However, many tumor cells exhibit a metabolic phenotype in which glucose uptake and lactate production remain elevated despite adequate oxygen availability, a phenomenon known as the Warburg effect (27, 28). This does not mean that mitochondrial metabolism is absent; rather, cancer cells often use aerobic glycolysis as a flexible strategy to rapidly generate ATP, maintain redox balance, and provide intermediates for biosynthetic pathways that support cell proliferation (29). In ovarian cancer, this glycolytic preference is better viewed as a dynamic metabolic adaptation than as a complete replacement of oxidative metabolism. From a functional perspective, the Warburg effect provides multiple advantages to malignant cells. Increased glycolytic throughput supports the synthesis of nucleotides, amino acids, and lipids required for biomass accumulation, while lactate secretion acidifies the extracellular space and contributes to a microenvironment that favors invasion, stromal remodeling, and immune dysfunction. In addition, routing pyruvate away from mitochondrial oxidation can help tumor cells tolerate hypoxia, detachment stress, and therapy-induced injury. Accordingly, glycolysis in cancer should be understood not merely as an inefficient way to produce energy, but as a central adaptive program that integrates metabolism with proliferation, survival, and microenvironmental interaction.

### 2.2 Key glycolytic phenotypes observed in ovarian cancer

A recurring feature of ovarian cancer is enhanced glucose uptake, most prominently through upregulation of GLUT1. Experimental work has shown that ovarian cancer cells depend strongly on GLUT1 to sustain both basal and stress-induced glycolysis, and pharmacologic inhibition with BAY-876 markedly reduces glycolytic activity, lactate production, anchorage-dependent and anchorage-independent growth, and *in vivo* tumorigenicity. These findings indicate that increased glucose transport is not simply a correlative marker but a functional requirement for the glycolytic phenotype in ovarian cancer.

Another prominent feature is the high expression of HK2, which catalyzes the first committed intracellular step of glucose metabolism. In epithelial ovarian tumors, HK2 expression is significantly higher than in normal ovarian tissue, benign ovarian tumors, and borderline lesions at both the protein and mRNA levels (30–33). Increased HK2 expression has also been associated with advanced stage, poorer differentiation, and serous histology, suggesting that early reinforcement of glycolytic flux is linked to more aggressive disease biology (34). Functionally, HK2 is not only a metabolic enzyme but also a contributor to malignant phenotypes including migration, invasion, stem-like traits, and resistance to therapeutic stress.

At the level of glycolytic commitment and amplification, PFKFB3 has emerged as another key node in ovarian cancer. By increasing fructose-2, 6-bisphosphate and thereby stimulating PFK1 activity, PFKFB3 enhances glycolytic throughput and supports rapid metabolic adaptation. Recent studies indicate that PFKFB3 is closely linked to ovarian cancer cell proliferation, metastasis, stemness, and chemoresistance, underscoring that control of the mid-glycolytic checkpoint has broad phenotypic consequences (35, 36). In parallel, PDK1 contributes to the glycolytic state by phosphorylating and inhibiting pyruvate dehydrogenase, thereby restricting pyruvate entry into mitochondrial oxidation and favoring its diversion toward lactate-generating pathways. This shift reinforces the Warburg phenotype by functionally decoupling glucose catabolism from full oxidative metabolism.

A further hallmark of glycolytic reprogramming in ovarian cancer is increased LDHA-dependent lactate production and the accumulation of lactate within the tumor milieu. Elevated lactate output is not metabolically neutral. It contributes to extracellular acidification, facilitates matrix remodeling and invasive behavior, and promotes paracrine signaling that can reshape stromal and immune components of the tumor microenvironment. Thus, the typical glycolytic phenotype in ovarian cancer includes not only enhanced glucose import and utilization, but also a pronounced metabolic endpoint characterized by lactate accumulation and microenvironmental acidification. Taken together, GLUT1 upregulation, HK2 overexpression, PFKFB3 activation, PDK1-mediated restriction of pyruvate oxidation, LDHA-driven lactate generation, and acidified extracellular conditions represent the major glycolytic traits repeatedly observed in ovarian cancer models and tissues.

### 2.3 Evidence supporting glycolytic dependency in ovarian cancer

Evidence for glycolytic dependency in ovarian cancer has accumulated across cellular, tissue, and animal model systems. At the cellular level, multiple studies have shown that inhibiting glycolytic nodes produces clear anti-tumor effects (37–40). Selective blockade of GLUT1 with BAY-876 suppresses glucose metabolism and substantially impairs ovarian cancer cell growth under both adherent and non-adherent conditions, indicating that glycolysis is required not only for proliferation but also for survival under stress. Similarly, targeting HK2, PFKFB3, or related metabolic regulators has been reported to reduce proliferation, migration, stem-like properties, and resistance-associated phenotypes, supporting the concept that ovarian cancer cells are not merely glycolytic in appearance but are functionally dependent on this program (41).

Tissue-level observations further reinforce this conclusion. Clinical specimen analyses have demonstrated that glycolytic enzymes such as HK2 are overexpressed in malignant ovarian tissues relative to non-malignant controls, and that higher expression correlates with features of more advanced or poorly differentiated disease. These associations suggest that glycolytic activation is not an incidental *in vitro* artifact but a clinically relevant characteristic of ovarian tumor biology. In addition, broader experimental studies evaluating glycolytic enzymes in ovarian cancer have supported the idea that the pathway is frequently upregulated and may represent a therapeutically actionable vulnerability.

**Figure 1:**
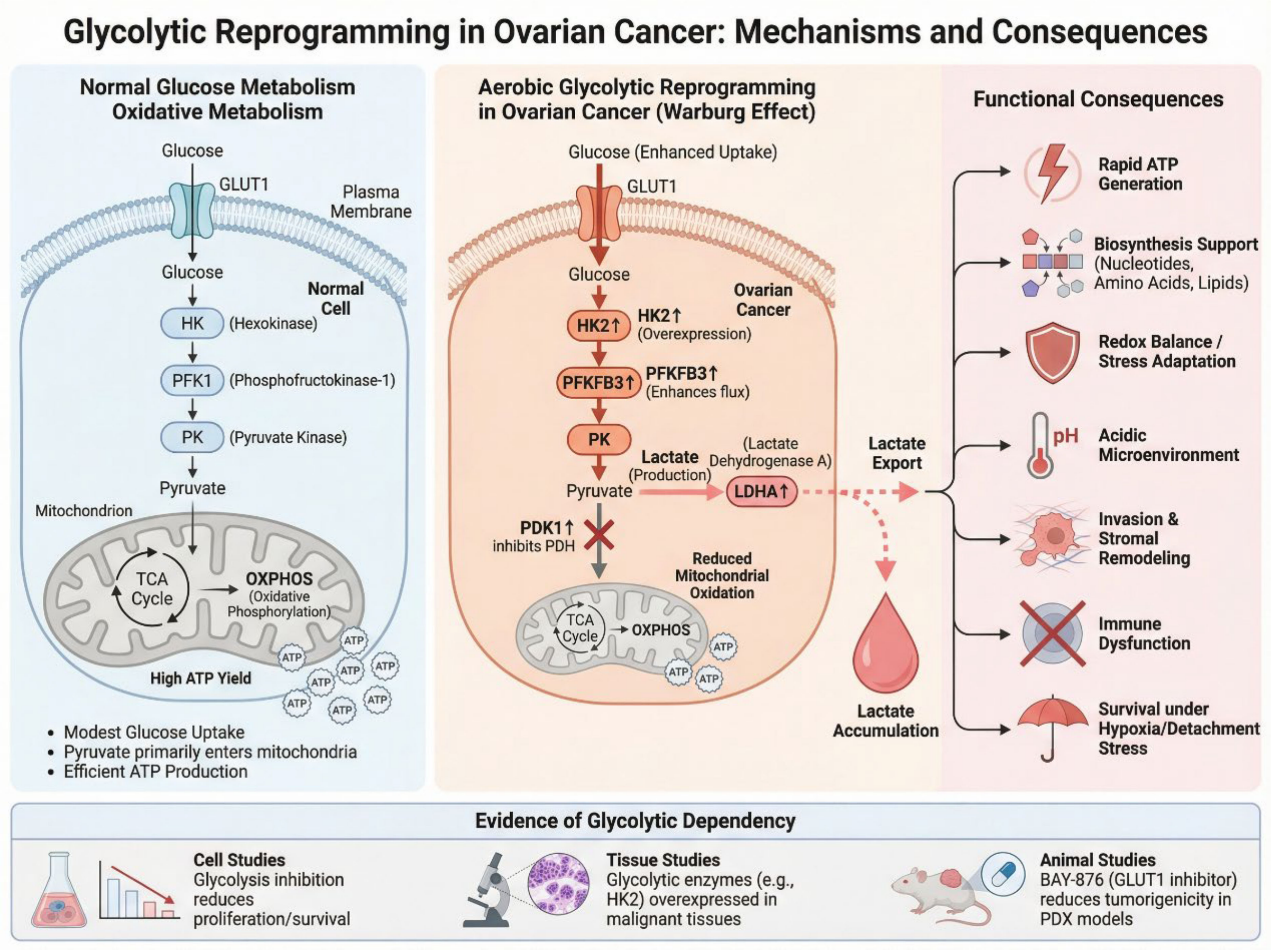
Overview of glycolytic reprogramming and glycolytic dependency in ovarian cancer. Ovarian cancer exhibits enhanced glucose uptake and aerobic glycolysis characterized by GLUT1, HK2, PFKFB3, PDK1, and LDHA dysregulation. These changes promote lactate accumulation, extracellular acidification, biosynthetic support, and stress adaptation while reducing pyruvate oxidation. Experimental evidence from cell, tissue, and animal studies supports that ovarian cancer is functionally dependent on glycolytic reprogramming.

Animal studies provide especially strong support for a genuine metabolic dependency. In xenograft and patient-derived xenograft models, inhibition of GLUT1 with BAY-876 dramatically reduces tumorigenicity, offering direct *in vivo* evidence that ovarian tumors can be constrained by disrupting glucose uptake (42). The fact that suppression of a single transporter can significantly impair tumor growth indicates that glycolysis occupies a non-redundant position in at least a substantial subset of ovarian cancers. Collectively, the convergence of mechanistic cell studies, clinicopathologic tissue analyses, and *in vivo* therapeutic experiments supports the conclusion that ovarian cancer frequently exhibits a meaningful dependence on glycolytic reprogramming. This dependency provides the basis for examining, in greater depth, the upstream molecular mechanisms that drive glycolysis and the downstream consequences for progression, immune remodeling, and therapeutic response. **Figure 1** illustrates the major features of glycolytic reprogramming in ovarian cancer, highlighting increased glucose uptake, reinforced glycolytic flux, restricted pyruvate oxidation, and enhanced lactate production. It also summarizes the downstream consequences of this metabolic state and the experimental evidence supporting glycolytic dependency as a therapeutic vulnerability in ovarian cancer.

## 3 Molecular mechanisms driving glycolytic reprogramming in ovarian cancer

### 3.1 Glucose transport and the initiation of glycolytic flux

The initiation of glycolytic reprogramming in ovarian cancer begins with enhanced glucose transport across the plasma membrane. Among the facilitative glucose transporters, GLUT1 has emerged as the dominant mediator of glucose influx in ovarian cancer and a key determinant of glycolytic substrate availability. Increased GLUT1 expression and activity allow tumor cells to maintain high intracellular glucose supply under both basal conditions and microenvironmental stress, thereby sustaining the elevated glycolytic flux required for rapid proliferation and survival. Functionally, GLUT1 should therefore be viewed not merely as a passive transporter, but as a gatekeeping node that sets the metabolic capacity of the glycolytic pathway in ovarian cancer (43–46). Direct experimental evidence supports this concept. In ovarian cancer cell models, selective pharmacologic inhibition of GLUT1 with BAY-876 markedly suppresses glucose utilization, reduces lactate production, and impairs both anchorage-dependent and anchorage-independent growth. The latter observation is particularly important because ovarian cancer cells must often survive in detached states during peritoneal dissemination. *In vivo*, BAY-876 significantly reduces tumorigenicity in both xenograft and patient-derived xenograft models, indicating that GLUT1-mediated glucose uptake is not simply associated with tumor growth but is mechanistically required for it. These findings strongly support the idea that enhanced glucose transport constitutes an early and functionally critical step in ovarian cancer glycolytic reprogramming.

**Figure 2:**
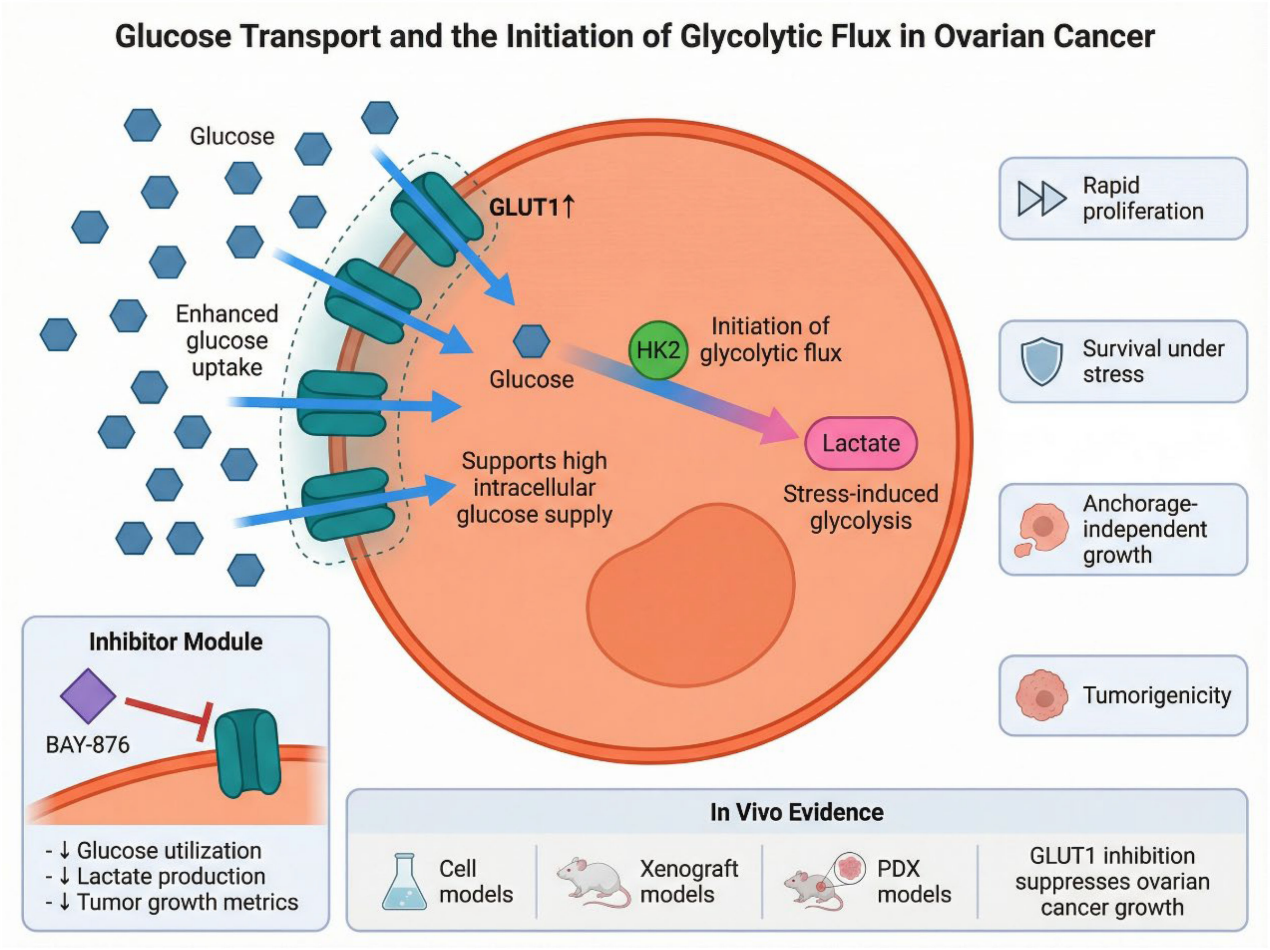
GLUT1-mediated initiation of glycolytic flux in ovarian cancer. Upregulated GLUT1 enhances glucose uptake and sustains intracellular glucose supply, thereby initiating and supporting glycolytic reprogramming in ovarian cancer. Increased glucose influx promotes lactate production, proliferation, and survival under stress. Pharmacologic inhibition of GLUT1 with BAY-876 suppresses glucose utilization, anchorage-dependent and anchorage-independent growth, and tumorigenicity in preclinical ovarian cancer models.

From a broader mechanistic perspective, increased GLUT1 activity also provides the foundation upon which downstream glycolytic enzymes can operate at high capacity. Even when oncogenic or microenvironmental signals activate HK2, PFKFB3, or LDHA, sustained glycolytic throughput remains limited unless sufficient glucose is imported into the cell. Thus, upregulated GLUT1 acts as the metabolic entry point that enables the full Warburg-like phenotype of ovarian cancer. This also explains why GLUT1 inhibition has such robust antitumor effects: it disrupts the pathway at its earliest supply step and thereby compromises multiple downstream malignant processes simultaneously (**Figure 2**).

### 3.2 Hexokinase-centered control of glycolysis

**Figure 3:**
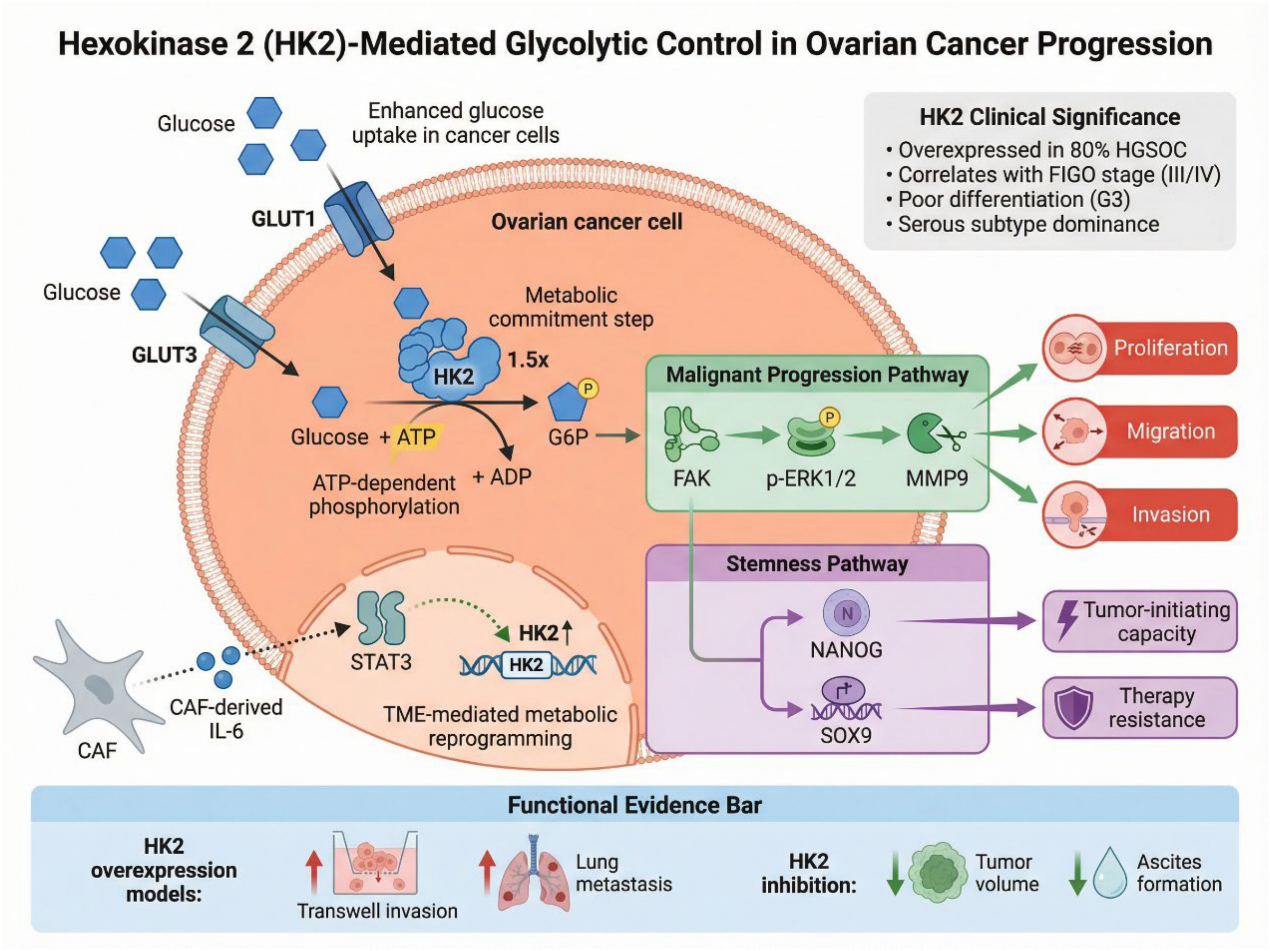
HK2-centered control of glycolysis in ovarian cancer. HK2 catalyzes glucose phosphorylation and commits intracellular glucose to glycolytic metabolism in ovarian cancer. HK2 overexpression is associated with advanced clinicopathologic features and promotes proliferation, migration, invasion, and stem-like properties through FAK/ERK1/2/MMP9 and FAK/ERK1/2/NANOG/SOX9 signaling. Stromal IL-6 further reinforces HK2 upregulation, linking glycolytic commitment to tumor–stromal crosstalk.

Following glucose entry, hexokinase 2 (HK2) occupies a central position in glycolytic control by catalyzing the phosphorylation of glucose to glucose-6-phosphate, thereby trapping glucose within the cell and committing it to intracellular metabolism. In ovarian cancer, HK2 is frequently overexpressed at both the mRNA and protein levels. Clinical specimen analyses have shown that HK2 expression is significantly higher in epithelial ovarian cancers than in normal ovarian tissue, benign tumors, or borderline lesions, and that elevated HK2 is associated with more advanced stage, poorer differentiation, and serous histology (47). These findings indicate that HK2 upregulation is not only a metabolic feature but also a clinically meaningful marker of aggressive disease biology. The role of HK2 in ovarian cancer extends well beyond its canonical enzymatic function. Functional studies have demonstrated that HK2 promotes tumor cell proliferation, migration, invasion, and cancer stem cell-like properties. HK2 overexpression enhances ovarian cancer cell motility and invasive capacity, whereas HK2 depletion suppresses these phenotypes and reduces *in vivo* tumor growth and dissemination. Mechanistically, these effects are linked to activation of the FAK/ERK1/2/MMP9 cascade, which couples metabolic activity to cytoskeletal remodeling, extracellular matrix interaction, and proteolytic invasion. In parallel, HK2 also promotes stemness through the FAK/ERK1/2/NANOG/SOX9 axis, suggesting that early glycolytic commitment is tightly connected to the maintenance of aggressive and plastic tumor cell states (48). An additional layer of complexity is that HK2 expression in ovarian cancer is responsive to microenvironmental cues. Ovarian cancer-associated fibroblast-derived IL-6 has been shown to contribute to HK2 upregulation, indicating that stromal signaling can reinforce glycolytic commitment (49). This observation is important because it places HK2 at the intersection of metabolism and tumor–stromal crosstalk. Taken together, the available evidence identifies HK2 as a central metabolic-effector molecule in ovarian cancer that links enhanced glycolysis to proliferative drive, invasive behavior, and stem-like plasticity. **Figure 3** illustrates how HK2 functions as a central metabolic-effector node in ovarian cancer by coupling early glycolytic commitment to proliferative, invasive, and stemness-associated programs. It also highlights that CAF-derived IL-6 can reinforce HK2 expression, placing HK2 at the interface between metabolic reprogramming and tumor–stromal crosstalk.

### 3.3 PFKFB3 and amplification of glycolytic commitment

Among the enzymes that reinforce glycolytic commitment, PFKFB3 has attracted particular attention because of its ability to amplify glycolytic throughput at a key regulatory checkpoint. PFKFB3 synthesizes fructose-2, 6-bisphosphate, a potent allosteric activator of PFK1, thereby promoting the irreversible commitment of carbon flux toward glycolysis. In ovarian cancer, PFKFB3 functions as a metabolic amplifier that enables sustained high-rate glycolysis under conditions of rapid growth and stress adaptation. Its role is therefore not limited to metabolic fine-tuning; rather, it helps lock tumor cells into a glycolysis-favoring state.

**Figure 4:**
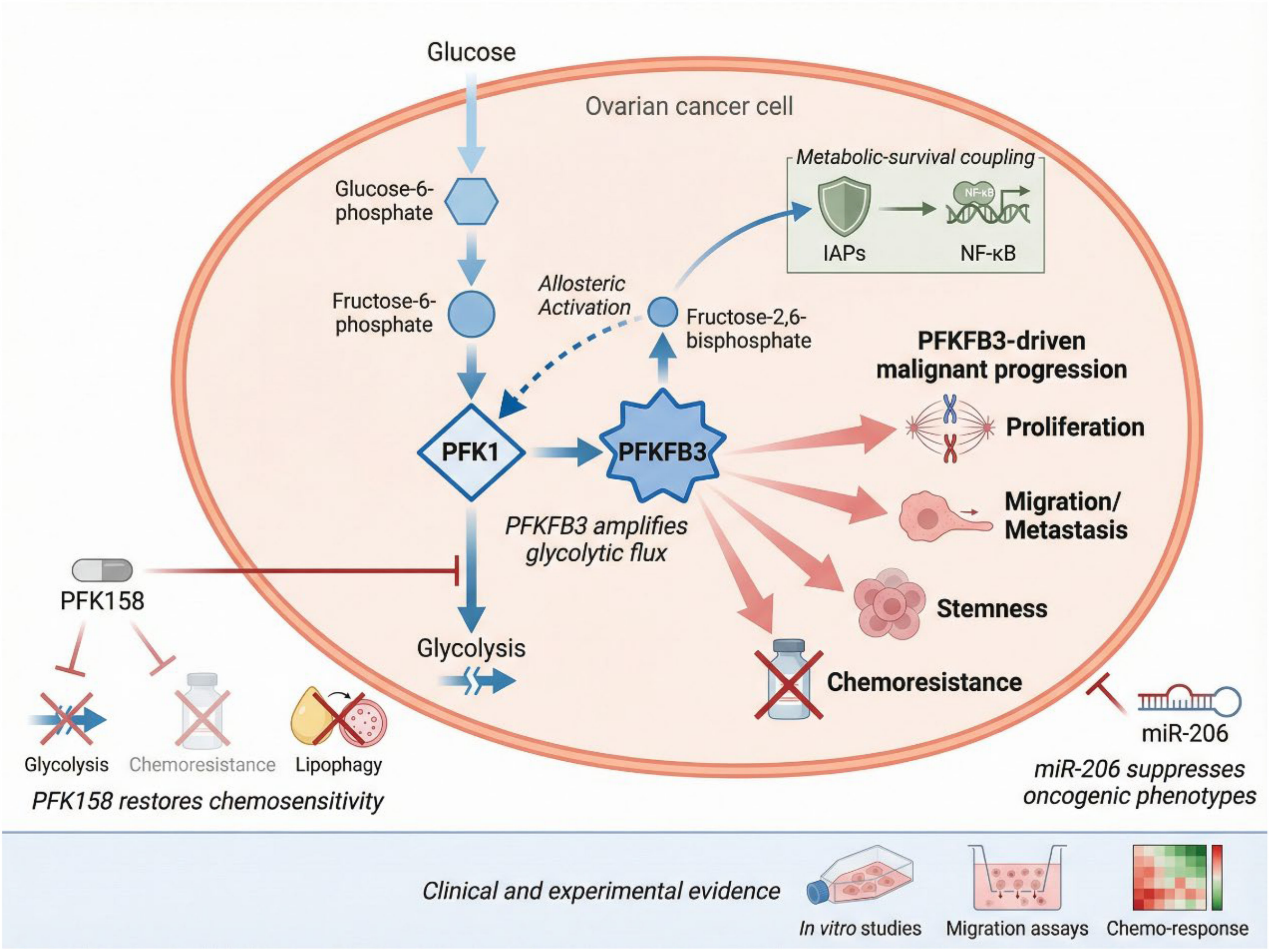
PFKFB3 amplifies glycolytic commitment in ovarian cancer. PFKFB3 enhances glycolytic flux by generating fructose-2, 6-bisphosphate and activating PFK1, thereby reinforcing glycolytic commitment. Increased PFKFB3 is linked to proliferation, metastasis, stemness, and chemoresistance in ovarian cancer, partly through survival-associated signaling such as IAPs and NF-κB. Pharmacologic inhibition with PFK158 or suppression by miR-206 attenuates malignant phenotypes and increases chemosensitivity.

Experimental studies have shown that PFKFB3 is closely linked to malignant progression in ovarian cancer. Increased PFKFB3 expression is associated with cell proliferation, migration, metastasis, stemness, and chemoresistance. Mechanistically, these effects have been linked to modulation of inhibitor of apoptosis proteins (IAPs) and the NF-κB signaling pathway, suggesting that PFKFB3 integrates metabolic flux with survival and stress-response signaling. This is particularly important in the setting of ovarian cancer, where tumor cells must adapt to both cytotoxic therapy and hostile microenvironmental conditions. In this context, PFKFB3 serves as a bridge between metabolic commitment and durable malignant fitness. Pharmacologic inhibition studies further underscore the therapeutic relevance of this enzyme. The PFKFB3 inhibitor PFK158 has been reported to suppress glycolysis and promote lipophagy while enhancing chemosensitivity in gynecologic cancer models, supporting the view that interference with glycolytic commitment can destabilize adaptive tumor programs. In addition, miRNA-based evidence strengthens this concept: miR-206 inhibits ovarian cancer cell proliferation and migration by targeting PFKFB3, with accompanying changes in glycolysis-related proteins such as GLUT1 and downstream signaling elements including FAK (50). Collectively, these observations position PFKFB3 as both a mechanistic driver and a translationally attractive vulnerability in ovarian cancer glycolytic reprogramming (**Figure 4**).

### 3.4 Pyruvate fate remodeling: the role of PDK1

**Figure 5:**
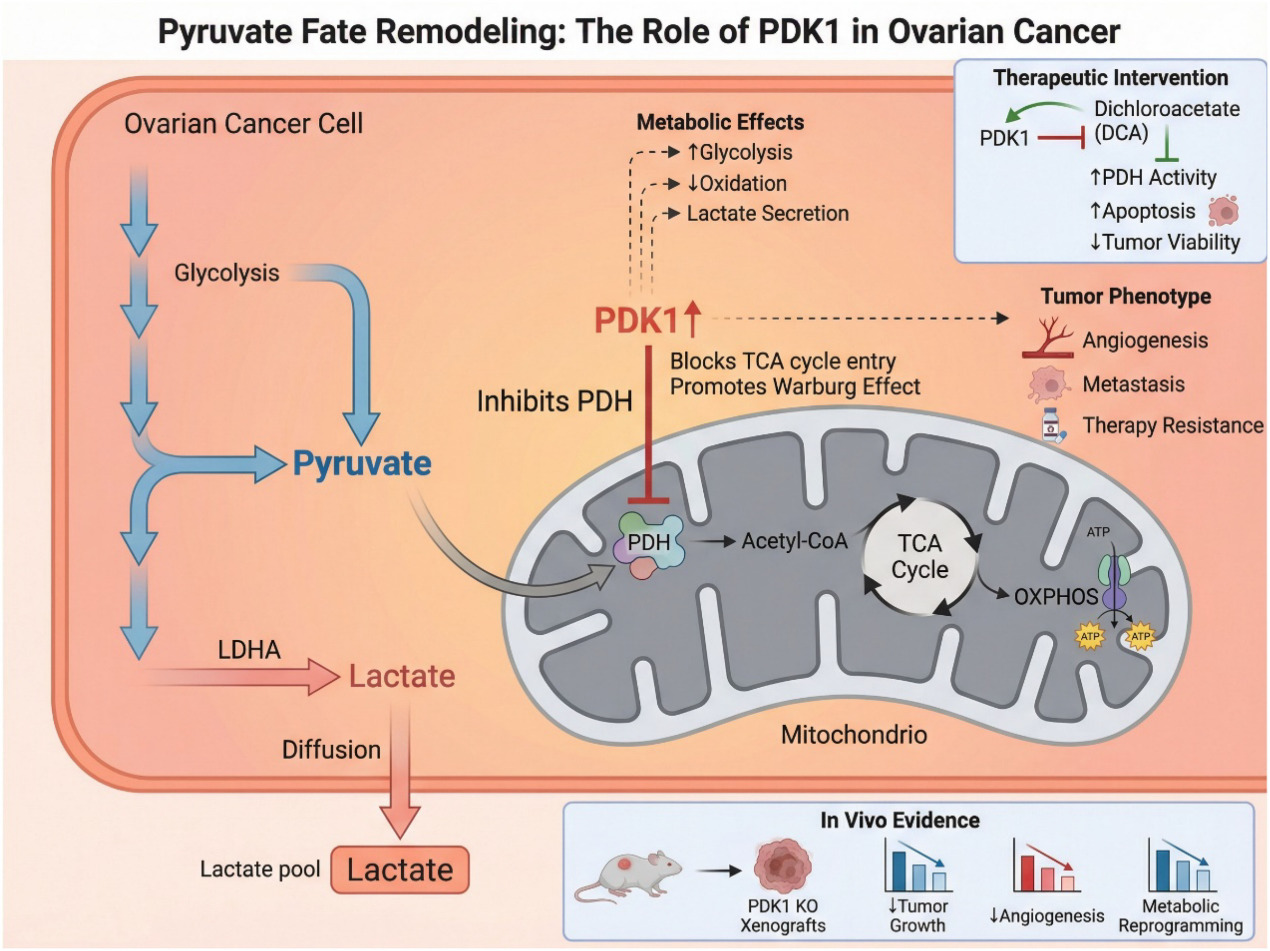
PDK1 redirects pyruvate metabolism in ovarian cancer. PDK1 phosphorylates and inhibits PDH, thereby restricting pyruvate entry into mitochondrial oxidation and favoring diversion toward lactate-producing pathways. This metabolic shift reinforces the Warburg phenotype and supports tumor growth, angiogenesis, metastasis, and therapy resistance. Pharmacologic targeting with dichloroacetic acid (DCA) may restore pyruvate oxidation, improve mitochondrial function, and increase apoptosis in ovarian cancer cells.

A defining feature of the Warburg phenotype is not only accelerated glucose breakdown but also altered handling of the end product, pyruvate. Pyruvate dehydrogenase kinase 1 (PDK1) plays a central role in this process by phosphorylating and inhibiting pyruvate dehydrogenase, thereby preventing pyruvate from entering the tricarboxylic acid cycle as acetyl-CoA. By restricting mitochondrial oxidation and preserving pyruvate for non-oxidative metabolism, PDK1 shifts carbon flow away from oxidative phosphorylation and toward lactate production. In ovarian cancer, this mechanism contributes directly to the maintenance of aerobic glycolysis and metabolic plasticity (51). Experimental evidence indicates that PDK1 is functionally important for ovarian cancer growth and progression (52). Genetic silencing of PDK1 in ovarian cancer xenograft models alters metabolic pathways, reduces glycolytic features, and significantly affects tumor growth and angiogenesis (53). These *in vivo* findings are particularly valuable because they move beyond cell culture observations and demonstrate that pyruvate fate remodeling contributes to tumor maintenance in a physiologic tumor context. Moreover, separate work has shown that PDK1 promotes ovarian cancer metastasis by modulating tumor–mesothelial adhesion, invasion, angiogenesis, and lactate production, further linking pyruvate metabolism to dissemination-related phenotypes. PDK1 is also relevant in the context of therapy response. By limiting mitochondrial oxidation, PDK1 can help cancer cells avoid excessive oxidative stress and maintain survival under cytotoxic challenge, thereby reinforcing chemoresistance. This has led to interest in PDK1-targeting strategies such as dichloroacetic acid, which aim to reverse the glycolytic phenotype and force greater reliance on mitochondrial metabolism. Overall, PDK1 serves as a pivotal controller of pyruvate fate in ovarian cancer, enabling the metabolic architecture of the Warburg effect while also promoting tumor growth, angiogenesis, metastasis, and treatment adaptation (**Figure 5**).

### 3.5 Lactate production and LDHA-mediated metabolic output

At the distal end of glycolysis, LDHA catalyzes the conversion of pyruvate to lactate while regenerating NAD+, thereby sustaining continuous glycolytic flux. In ovarian cancer, LDHA is more than a terminal metabolic enzyme; it is a major determinant of the biochemical output and biological consequences of glycolytic reprogramming (54–57). Elevated LDHA activity supports high lactate production, allowing tumor cells to maintain redox balance and preserve glycolytic throughput under conditions of rapid metabolic demand. The functional significance of LDHA in ovarian cancer has been demonstrated by studies showing that manipulation of miR-383 alters glycolysis, proliferation, and invasion through direct targeting of LDHA. miR-383 expression is negatively correlated with LDHA in ovarian cancer tissues, and restoration of miR-383 suppresses aerobic glycolysis as well as malignant phenotypes, whereas LDHA overexpression reverses these effects (58). These findings support a causal role for LDHA in promoting ovarian cancer progression and confirm that lactate-generating output is integral to the oncogenic consequences of glycolytic reprogramming.

**Figure 6:**
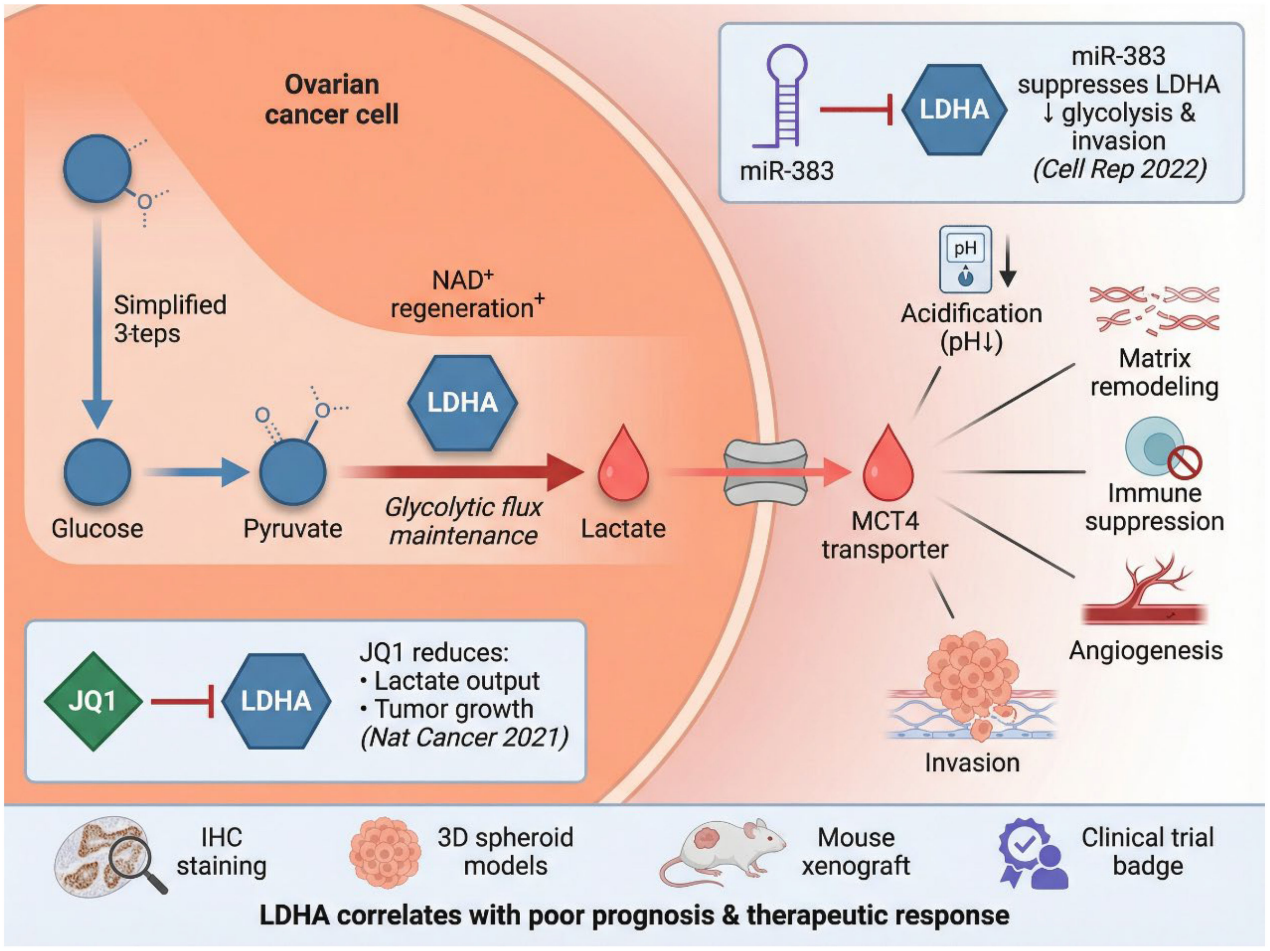
LDHA-driven metabolic output in ovarian cancer. LDHA converts pyruvate to lactate, regenerates NAD+, and sustains glycolytic flux in ovarian cancer. Increased lactate production promotes extracellular acidification, matrix remodeling, invasive behavior, paracrine signaling, and immune dysfunction. Suppression of LDHA by miR-383 or pharmacologic inhibition such as JQ1 reduces glycolysis-associated malignant phenotypes and supports LDHA as a therapeutic target.

Lactate production itself also has broad pathobiological implications. Increased lactate accumulation contributes to extracellular acidification, which can promote invasion, matrix remodeling, angiogenic signaling, and immune dysfunction. Although much of the literature in ovarian cancer has focused on tumor cell-intrinsic effects, growing evidence suggests that lactate also participates in tumor–stroma and tumor–immune communication, helping to create a permissive microenvironment for progression and immune escape. From a therapeutic standpoint, these observations make LDHA an attractive intervention point: targeting LDHA has the potential not only to reduce glycolytic flux but also to mitigate the downstream protumorigenic consequences of lactate accumulation (**Figure 6**).

### 3.6 Upstream oncogenic signaling pathways controlling glycolysis

The glycolytic phenotype of ovarian cancer is reinforced by a network of upstream oncogenic and microenvironment-responsive signaling pathways that converge on core glycolytic genes and enzymes (59, 60). Among these, the PI3K/AKT/HIF-1α axis is especially important. Activation of PI3K/AKT signaling stabilizes or enhances HIF-1α, which in turn transcriptionally upregulates major glycolytic genes and promotes a glycolysis-favoring transcriptional program. In ovarian cancer, SIK2 has been shown to enhance glucose metabolic reprogramming through PI3K/AKT/HIF-1α signaling while also promoting Drp1-mediated mitochondrial fission, thereby coupling increased glycolysis with reduced oxidative phosphorylation (61). This dual mechanism strongly reinforces the Warburg phenotype by both increasing glycolytic gene expression and functionally suppressing mitochondrial respiration.

Additional oncogenic regulators further intensify this metabolic state. MYC-associated signaling promotes glycolysis in ovarian cancer, and Pim1 has been reported to regulate glycolysis through interaction with MYC, linking growth signaling to metabolic activation (62). Wnt/β-catenin signaling also contributes to glycolytic control, as evidenced by studies showing that HK2 can promote ovarian cancer progression through Wnt/β-catenin-dependent upregulation of proliferative effectors such as cyclin D1 and c-Myc (63). Conversely, tumor suppressive mechanisms can restrain glycolytic reprogramming. In particular, p53 has been shown to promote chemosensitivity in epithelial ovarian cancer by regulating HKII gene transcription and metabolic reprogramming, highlighting that altered p53 status may contribute to glycolytic adaptation and treatment resistance.

**Figure 7:**
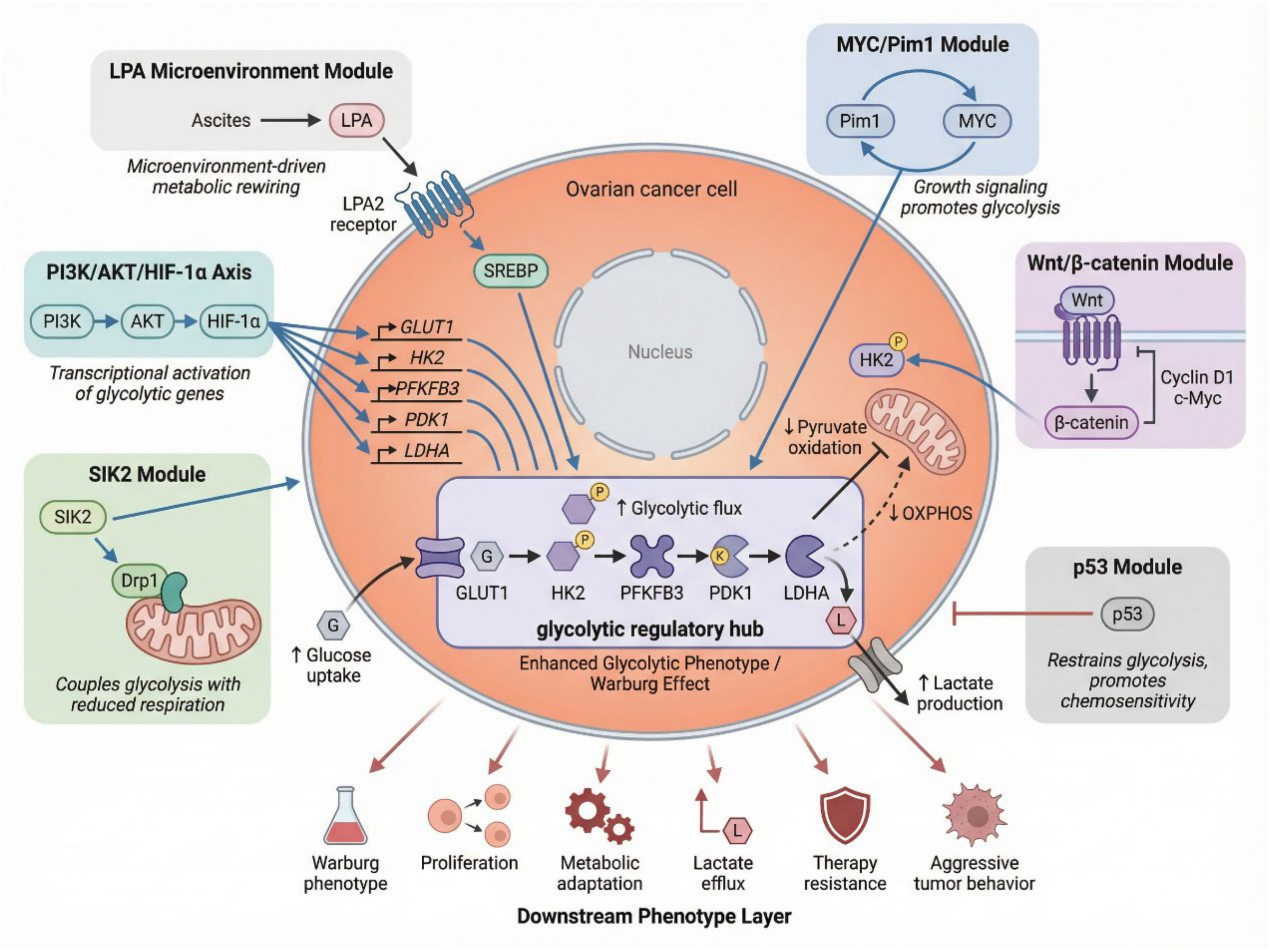
Upstream signaling pathways controlling glycolytic reprogramming in ovarian cancer. Oncogenic and microenvironment-responsive pathways, including PI3K/AKT/HIF-1α, SIK2, MYC/Pim1, Wnt/β-catenin, p53, and LPA/LPA2/SREBP, converge on core glycolytic regulators such as GLUT1, HK2, PFKFB3, PDK1, and LDHA. These integrated signals enhance glycolytic flux, promote the Warburg effect, reduce oxidative metabolism, and support aggressive tumor behavior and therapy resistance.

Microenvironment-derived signaling provides another important layer of regulation. Lysophosphatidic acid (LPA), a lipid mediator enriched in ovarian cancer ascites, has been shown to upregulate HK2 and increase glycolytic rate and lactate efflux in ovarian cancer cells (64). Mechanistically, this effect depends on LPA2, an LPA receptor subtype frequently overexpressed in ovarian cancer, and is mediated through sterol regulatory element-binding proteins at the HK2 promoter. This finding is especially noteworthy because it demonstrates how extracellular signals within the ovarian cancer microenvironment can directly reshape tumor cell metabolism. Taken together, pathways involving PI3K/AKT/HIF-1α, MYC, Wnt/β-catenin, p53, LPA-related signaling, and SIK2 do not operate in isolation; rather, they converge to drive the expression or activity of GLUT1, HK2, PFKFB3, LDHA, and PDK1, thereby establishing and maintaining the glycolytic phenotype of ovarian cancer (**Figure 7**).

### 3.7 Non-coding RNA-mediated regulation of glycolytic reprogramming

In addition to protein-coding oncogenic pathways, non-coding RNAs (ncRNAs) constitute an important post-transcriptional regulatory layer that coordinates glycolytic reprogramming in ovarian cancer (50, 65). Rather than acting as isolated regulators, ncRNAs form an interconnected network that modulates glycolytic enzyme expression, pyruvate fate, lactate production, mitochondrial metabolism, and upstream oncogenic signaling. This network provides a flexible mechanism through which ovarian cancer cells fine-tune metabolic flux in response to intrinsic oncogenic stress and microenvironmental cues.

Mechanistically, ncRNA-mediated regulation of glycolysis can be organized into three major modules. First, some miRNAs directly target key glycolytic enzymes and thereby control glycolytic throughput. For example, miR-383 suppresses aerobic glycolysis, proliferation, and invasion by targeting LDHA, reducing lactate-generating output (58). Similarly, miR-206 targets PFKFB3, limiting fructose-2, 6-bisphosphate production, glycolytic commitment, and malignant phenotypes (50). These findings suggest that miRNA-dependent repression of glycolytic enzymes can function as a metabolic brake that restrains ovarian cancer aggressiveness.

Second, ncRNAs regulate the balance between glycolysis and mitochondrial oxidation by controlling pyruvate fate. miR-203 promotes ovarian cancer cell growth and migration by suppressing PDHB, a component of the pyruvate dehydrogenase complex (66). Through this mechanism, miR-203 reduces pyruvate entry into mitochondrial oxidative metabolism and favors a glycolysis-dominant state. This illustrates that ncRNAs can promote glycolytic reprogramming not only by regulating canonical glycolytic enzymes, but also by redirecting carbon flux away from mitochondrial oxidation.

Third, lncRNAs can amplify glycolytic reprogramming through competing endogenous RNA (ceRNA)-like mechanisms and broader metabolic network control. LINC00504 promotes ovarian cancer progression and stimulates the Warburg effect through inhibition of miR-1244, suggesting that lncRNA–miRNA interactions can indirectly sustain glycolytic activation (67). SNHG3 further expands this regulatory model, as proteomic analyses indicate that it influences mitochondrial and energy metabolism programs relevant to ovarian cancer progression (68). Thus, lncRNAs may function as network-level regulators that connect glycolytic flux with mitochondrial remodeling and tumor cell adaptability.

**Figure 8:**
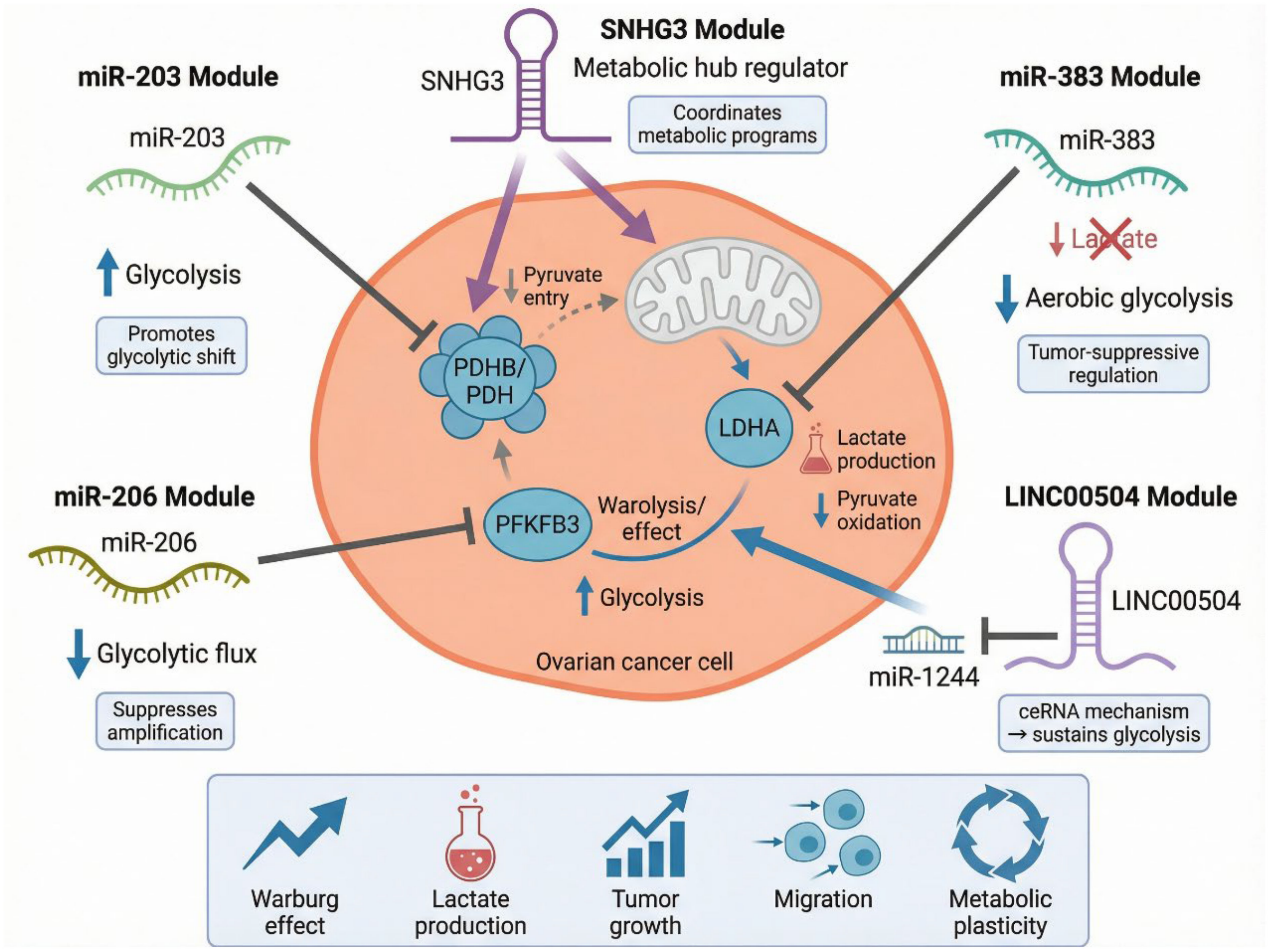
Non-coding RNA-mediated regulation of glycolytic reprogramming in ovarian cancer. Non-coding RNAs regulate ovarian cancer glycolysis by targeting key metabolic nodes and mitochondrial programs. miR-203 promotes glycolysis by suppressing PDHB, whereas miR-383 and miR-206 inhibit glycolytic output by targeting LDHA and PFKFB3, respectively. LINC00504 and SNHG3 further sustain glycolytic reprogramming through ceRNA-like and broader metabolic regulatory mechanisms, thereby supporting malignant progression.

Taken together, ncRNAs should be viewed as central components of a multilayered glycolytic regulatory network in ovarian cancer. Through direct control of glycolytic enzymes, modulation of pyruvate metabolism, and ceRNA-mediated amplification of metabolic signaling, ncRNAs connect post-transcriptional regulation to tumor progression, chemoresistance, and immune escape. This integrated perspective moves beyond individual ncRNA–target pairs and highlights ncRNA networks as potential biomarkers and therapeutic entry points for metabolic intervention in ovarian cancer. **Table 1** summarizes representative experimental studies defining the major molecular drivers of glycolytic reprogramming in ovarian cancer, with particular emphasis on GLUT1, HK2, PFKFB3, PDK1, LDHA, and their associated signaling networks (**Figure 8**).

**Table 1:** Representative experimental studies on glycolytic reprogramming in ovarian cancer.

Study	Year	Target/pathway	Model	Main findings	Relevance
Ma et al.	2019	GLUT1/BAY-876	Ovarian cancer cell lines, xenografts, PDXs	Ovarian cancer depends on GLUT1 for basal and stress-regulated glycolysis; BAY-876 reduced lactate production, impaired anchorage-dependent and independent growth, and suppressed tumorigenicity	Strong evidence that glucose transport is a functional metabolic vulnerability
Jin et al. (47)	2014	HK2	Human ovarian tissue samples	HK2 expression was significantly increased in epithelial ovarian cancer and associated with advanced stage, poorer differentiation, and serous histology	Supports the clinical relevance of HK2 upregulation
Siu et al. (48)	2019	HK2/FAK–ERK1/2–MMP9/NANOG–SOX9	Ovarian cancer cells, xenografts	HK2 promoted proliferation, migration, invasion, and stemness; linked glycolysis to FAK/ERK signaling, MMP9, NANOG, and SOX9	Mechanistically connects glycolysis to aggressive phenotypes
Mondal et al. (69)	2019	PFKFB3/PFK158	Gynecologic cancer models including ovarian cancer	PFK158 inhibited glycolysis, promoted lipophagy, and enhanced chemosensitivity	Highlights PFKFB3 as a therapeutic target
Jiang et al. (70)	2022	PFKFB3	Ovarian cancer cells and tumor models	PFKFB3 regulated chemoresistance, metastasis, and stemness; linked to survival signaling	Positions PFKFB3 as a central glycolytic amplifier in progression
Venturoli et al. (53)	2021	PDK1	Xenograft models	Genetic perturbation of PDK1 altered metabolic pathways, reduced tumor growth, and affected angiogenesis	Demonstrates the importance of pyruvate fate remodeling *in vivo*
Zhou et al. (113)	2019	PDK axis/Dichloroacetic acid	Ovarian cancer cells	DCA regulated glucose metabolism, restored mitochondrial function, and increased apoptosis	Supports targeting pyruvate metabolism to reverse Warburg-like adaptation
Han et al. (58)	2017	LDHA/miR-383	Ovarian cancer cells, tissues	miR-383 inhibited proliferation, invasion, and aerobic glycolysis by directly targeting LDHA	Establishes LDHA as a functional driver of malignant glycolytic output
Qiu et al. (71)	2015	LDHA/JQ1	Ovarian cancer cells and tumor models	JQ1 suppressed tumor growth through downregulation of LDHA	Suggests lactate-generating output is therapeutically actionable
Gao et al. (61)	2020	SIK2/PI3K–AKT–HIF-1α/Drp1	Ovarian cancer cells	SIK2 promoted glucose metabolic reprogramming and mitochondrial fission, favoring glycolysis and aggressive behavior	Integrates glycolysis with signaling and mitochondrial remodeling

## 4 Glycolysis as a driver of ovarian cancer progression

### 4.1 Glycolysis supports rapid proliferation and survival

Glycolytic reprogramming provides ovarian cancer cells with a growth advantage by supporting both energetic and biosynthetic demands. Although glycolysis yields less ATP per glucose molecule than oxidative phosphorylation, it can proceed rapidly and continuously, thereby furnishing sufficient energy for proliferating tumor cells while simultaneously supplying intermediates for nucleotide, amino acid, and lipid synthesis. This is especially advantageous in ovarian cancer, where tumor cells often face fluctuating oxygen and nutrient availability within the peritoneal cavity (74–76). By maintaining high glucose uptake and sustained glycolytic flux, ovarian cancer cells are able to support biomass accumulation, preserve redox balance, and survive under stress conditions that would otherwise compromise proliferation (77–80). Experimental evidence supports a direct link between glycolysis and ovarian cancer cell viability. Inhibition of GLUT1 with BAY-876 reduces glucose utilization, suppresses lactate production, and markedly impairs ovarian cancer cell growth under both adherent and anchorage-independent conditions, indicating that glucose-fueled metabolism is required not only for expansion in conventional culture but also for survival in stress-associated detached states. Similarly, enzymes such as HK2 and PFKFB3 are not merely markers of high metabolic activity; their depletion or inhibition leads to decreased proliferative capacity, suggesting that ovarian cancer cells are functionally dependent on glycolytic throughput for sustained growth. This survival-promoting role of glycolysis also reflects its integration with broader adaptive programs. By channeling carbon away from complete mitochondrial oxidation, ovarian cancer cells can reduce excessive oxidative burden while preserving metabolic flexibility. In this sense, glycolysis serves as a platform for stress adaptation rather than simply an alternative energy pathway. The ability of glycolytic regulators such as SIK2 and SNHG3 to influence broader energy metabolism further supports the view that glycolysis contributes to a coordinated survival network that helps ovarian cancer cells endure microenvironmental fluctuations and therapeutic injury.

### 4.2 Ovarian cancer-specific metabolic contexts: peritoneal dissemination, ascites, and anchorage-independent survival

A key feature that distinguishes ovarian cancer from many other solid tumors is its predominant transcoelomic dissemination within the peritoneal cavity. In advanced ovarian cancer, tumor cells detach from the primary lesion, survive as spheroids in suspension, interact with ascitic fluid, adhere to mesothelial surfaces, and establish peritoneal or omental implants, leading to peritoneal carcinomatosis and poor clinical outcome (81, 82). These steps expose cancer cells to unique metabolic pressures, including anchorage-independent stress, fluctuating oxygen tension, variable nutrient availability, and exposure to ascites-derived metabolites, cytokines, growth factors, and lipids in the malignant fluid (83). In this context, glycolytic reprogramming may provide a rapid and flexible energy source while supporting redox balance and survival under detachment-associated stress. Malignant ascites is also a metabolically active milieu with altered nutrient and metabolite profiles that reflect increased consumption and recycling by tumor and stromal cells (83). Furthermore, lactate accumulation and other ascites-derived factors contribute to extracellular acidification, immune suppression, and macrophage remodeling, shaping a peritoneal niche that supports tumor survival and immune escape. Therefore, glycolysis in ovarian cancer should be interpreted not only as a general Warburg-like phenotype but also as a disease-specific adaptive program that supports survival, dissemination, and colonization within the ascites-rich peritoneal ecosystem.

### 4.3 Glycolytic reprogramming promotes invasion and metastasis

A major consequence of glycolytic reprogramming in ovarian cancer is the acquisition of enhanced migratory and invasive behavior (84–87). This is particularly relevant in ovarian cancer because disease progression is characterized less by hematogenous spread in early stages and more by transcoelomic dissemination within the peritoneal cavity. For tumor cells to successfully detach, survive in suspension, adhere to mesothelial surfaces, and establish secondary lesions, they must coordinate metabolic adaptation with motility and matrix-remodeling programs. Glycolysis appears to support this process at multiple levels, including energy provision, cytoskeletal remodeling, extracellular acidification, and activation of invasion-related signaling cascades.

Among the strongest examples is HK2, which has been shown to promote ovarian cancer cell migration and invasion through the FAK/ERK1/2/MMP9 pathway. HK2 overexpression enhances these metastatic traits, whereas HK2 abrogation suppresses them and impedes *in vivo* tumor dissemination. These findings demonstrate that glycolytic control at the level of glucose phosphorylation can be coupled directly to cell adhesion signaling, extracellular matrix degradation, and motility (88). In other words, HK2 helps translate metabolic activation into invasive competence. Other glycolysis-related regulators reinforce this metastatic phenotype through complementary mechanisms. PFKFB3 has been linked to metastasis in ovarian cancer, consistent with its role in amplifying glycolytic commitment and maintaining high metabolic throughput. Likewise, SIK2 promotes reprogramming of glucose metabolism through the PI3K/AKT/HIF-1α pathway and Drp1-mediated mitochondrial fission, thereby enhancing a metabolic state favorable to aggressive tumor behavior. Collectively, these findings indicate that glycolysis in ovarian cancer is closely intertwined with dissemination-related plasticity rather than being confined to a purely metabolic phenotype.

### 4.4 Glycolysis and cancer stem-like properties

Increasing evidence suggests that glycolytic reprogramming also supports the maintenance of cancer stem-like properties in ovarian cancer (89). This is an important concept because stem-like tumor cells are thought to contribute disproportionately to recurrence, metastasis, and therapy failure. Glycolysis can favor stemness by sustaining rapid energy supply, preserving biosynthetic capacity, and allowing tumor cells to remain adaptable under environmental or therapeutic stress (90–92). In ovarian cancer, this metabolic state appears to be coupled to transcriptional programs that govern self-renewal and phenotypic plasticity.

The clearest evidence again comes from HK2, which has been shown to enhance stemness through the FAK/ERK1/2/NANOG/SOX9 signaling cascade. HK2 overexpression promotes stem cell-like features, whereas HK2 depletion suppresses them, linking glycolytic commitment directly to stemness-associated transcription factors. This finding is especially significant because it indicates that glycolysis is not merely permissive for stem-like behavior, but actively participates in the signaling circuitry that stabilizes aggressive cellular states. A similar pattern is observed with PFKFB3, which has been reported to regulate ovarian cancer stemness together with chemoresistance and metastasis. Since cancer stem-like cells often rely on adaptive metabolism to survive therapy and seed recurrence, the connection between PFKFB3-driven glycolysis and stemness helps explain why glycolytic activation is repeatedly associated with poor outcome-related phenotypes. These observations support the broader view that glycolysis contributes to clonal fitness and tumor plasticity by enabling a subset of ovarian cancer cells to occupy more aggressive, stem-like states.

### 4.5 Crosstalk between glycolysis and metabolic plasticity

**Figure 9:**
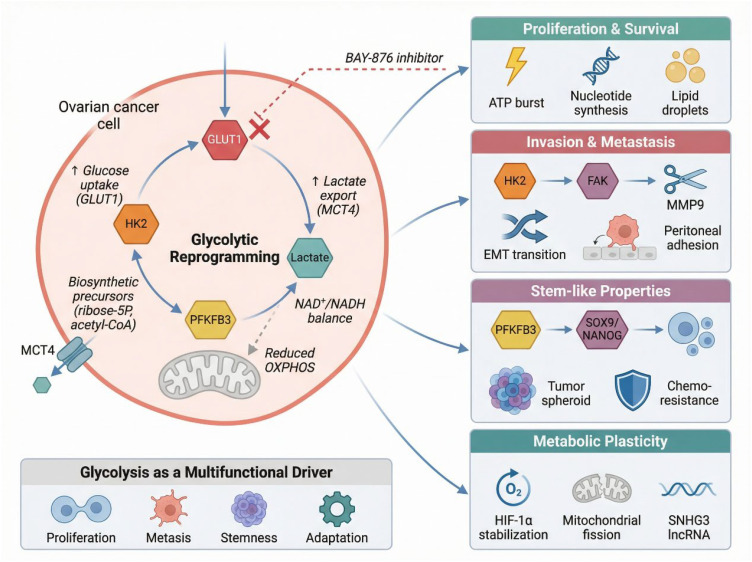
Glycolysis as a driver of ovarian cancer progression. Enhanced glycolysis supports ovarian cancer progression by promoting rapid proliferation, survival under stress, invasion, metastasis, stem-like properties, and metabolic plasticity. Key glycolytic regulators, including GLUT1, HK2, and PFKFB3, support biosynthesis, lactate production, and adaptive survival, while HK2-associated FAK/ERK1/2 signaling, SIK2, and SNHG3 connect glycolysis to dissemination, stemness, and broader metabolic remodeling.

Although glycolysis is a major metabolic feature of ovarian cancer, it does not operate in isolation. Instead, it is embedded within a broader network of metabolic plasticity, in which tumor cells dynamically rebalance glycolysis, mitochondrial metabolism, redox maintenance, and stress-response pathways according to environmental conditions (93). This flexibility is particularly important in ovarian cancer because tumor cells encounter highly variable contexts, including hypoxia, nutrient limitation, detachment stress, ascitic fluid exposure, and treatment-induced injury. Under these conditions, glycolysis functions as a central but adaptable metabolic axis rather than a fixed, exclusive state. Studies on SIK2 and SNHG3 highlight this point. SIK2 promotes glucose metabolic reprogramming through PI3K/AKT/HIF-1α signaling while simultaneously driving Drp1-mediated mitochondrial fission, indicating that ovarian cancer cells coordinate glycolytic activation with mitochondrial remodeling rather than simply switching mitochondria off (94–96). Meanwhile, SNHG3 regulates ovarian cancer energy metabolism at a broader systems level, as revealed by mitochondrial proteomic analyses, suggesting that non-coding RNA-mediated metabolic control extends beyond a single glycolytic enzyme to a more integrated energetic program (97). This crosstalk helps explain why glycolysis is so strongly associated with progression. By functioning within a flexible metabolic network, glycolysis allows ovarian cancer cells to maintain growth during favorable conditions while rapidly adapting to stress when conditions deteriorate. Such plasticity supports not only proliferation, invasion, and stemness, but also the emergence of treatment-tolerant states. Thus, glycolytic reprogramming should be regarded as a central driver of ovarian cancer progression precisely because it is embedded within a broader adaptive metabolic architecture. **Figure 9** illustrates how glycolytic reprogramming functions as a central driver of ovarian cancer progression by supporting proliferation, survival, invasion, metastasis, stem-like traits, and adaptive metabolic plasticity. It also highlights that glycolysis is tightly linked to signaling pathways such as HK2–FAK/ERK1/2, SIK2–PI3K/AKT/HIF-1α, and SNHG3-associated energy remodeling, thereby extending its impact beyond energy production alone.

## 5 Glycolysis and therapeutic resistance in ovarian cancer

### 5.1 Glycolysis as a metabolic basis of chemoresistance

Therapeutic resistance remains one of the most formidable barriers to durable disease control in ovarian cancer. Although many patients initially respond to platinum-based chemotherapy, relapse is common, and recurrent tumors frequently display an increased ability to survive cytotoxic stress. Accumulating evidence indicates that glycolytic reprogramming contributes importantly to this process by providing a metabolic framework that supports survival during treatment (98, 99). Rather than acting solely as an energy source, glycolysis helps ovarian cancer cells preserve ATP supply, maintain redox homeostasis, buffer oxidative stress, and limit mitochondrial damage under chemotherapeutic pressure. In this way, a glycolysis-dominant state enables tumor cells to better withstand therapy-induced injury and recover proliferative capacity after treatment.

This resistance-promoting effect is closely tied to the broader concept of metabolic plasticity. Chemotherapy imposes a severe stress environment in which tumor cells must simultaneously maintain membrane integrity, avoid apoptosis, and adapt to altered nutrient and oxygen conditions. Enhanced glycolysis can facilitate these demands by rapidly generating ATP and by routing intermediates into antioxidant and biosynthetic pathways. In ovarian cancer, such metabolic adaptation is especially relevant because resistant cells often coexist with detachment stress, hypoxia, ascitic fluid exposure, and stromal support, all of which can reinforce glycolytic dependency. Thus, glycolysis should be regarded as a central component of the adaptive survival program underlying chemoresistance rather than a secondary byproduct of aggressive growth (100–103). At a conceptual level, this also helps explain why glycolytic markers are repeatedly linked to poor-prognosis phenotypes. A tumor cell capable of sustaining high glycolytic throughput is better equipped to endure acute stress, maintain viability in hostile microenvironments, and transition into drug-tolerant states. Accordingly, metabolic rewiring toward glycolysis may not only accompany therapeutic resistance, but actively drive its emergence and stabilization in ovarian cancer.

### 5.2 HK2, PFKFB3, and PDK1 in drug resistance

Several glycolytic regulators have emerged as particularly important mediators of therapy resistance in ovarian cancer, including HK2, PFKFB3, and PDK1. Among these, HK2 occupies a prominent position because it governs the first committed step of intracellular glucose metabolism and integrates glycolytic activity with survival signaling. Functional evidence indicates that HK2 supports not only proliferation and invasion but also stem-like properties, which are themselves closely associated with resistance and recurrence (104, 105). By sustaining early glycolytic commitment, HK2 likely enables ovarian cancer cells to preserve metabolic resilience during chemotherapy and maintain a state conducive to post-treatment regrowth. PFKFB3 is another key resistance-associated node. As a potent amplifier of glycolytic commitment, PFKFB3 enhances fructose-2, 6-bisphosphate production and drives sustained glycolytic throughput. In ovarian cancer, PFKFB3 has been shown to regulate chemoresistance, metastasis, and stemness, indicating that it functions at the intersection of metabolic adaptation and malignant plasticity (70). Mechanistically, its association with IAP proteins and NF-κB signaling suggests that PFKFB3 may reinforce resistance not only by supporting metabolism, but also by promoting anti-apoptotic and stress-response pathways. This dual role makes PFKFB3 especially important in the context of ovarian cancer, where resistant cells must survive both direct cytotoxic damage and prolonged microenvironmental stress. The contribution of PDK1 to resistance derives from its control over pyruvate fate. By phosphorylating and inhibiting pyruvate dehydrogenase, PDK1 restricts pyruvate entry into mitochondrial oxidation and biases metabolism toward glycolysis and lactate production (106, 107). This reduces reliance on mitochondrial respiration and may help limit therapy-induced oxidative injury, thereby favoring cell survival. *In vivo* data showing that PDK1 perturbation alters tumor growth, angiogenesis, and metabolic pathways in ovarian cancer xenografts support the view that pyruvate fate remodeling is functionally important for therapy adaptation as well as tumor progression. Together, HK2, PFKFB3, and PDK1 exemplify how different control points within the glycolytic network cooperate to sustain drug-tolerant phenotypes in ovarian cancer.

### 5.3 p53-mediated metabolic control and chemosensitivity

The relationship between glycolysis and drug response in ovarian cancer is further illustrated by the role of p53, which can influence chemosensitivity through metabolic regulation. In epithelial ovarian cancer, p53 has been shown to promote chemosensitivity by regulating HKII gene transcription and metabolic reprogramming (108). This finding is notable because it directly links a canonical tumor suppressor pathway to a glycolytic effector and to therapeutic outcome. When p53-mediated control over glycolysis is intact, ovarian cancer cells may be less able to sustain the metabolic flexibility required for survival under chemotherapy. Conversely, disruption of this regulatory axis may favor a more glycolysis-dependent and therapy-resistant state. This p53–HK2 connection also highlights an important principle: the metabolic phenotype of ovarian cancer is not determined solely by classical metabolic enzymes, but also by upstream tumor suppressive and oncogenic networks that dictate how those enzymes are expressed and utilized. In resistant tumors, impaired p53 function may therefore facilitate glycolytic adaptation in a manner that extends beyond simple cell-cycle deregulation (109–111). Instead, it may contribute to the establishment of a metabolic state that supports survival, limits apoptosis, and reduces responsiveness to platinum-based agents. From a translational standpoint, the p53-regulated metabolic axis also suggests that metabolic vulnerabilities may differ according to molecular context. Tumors with defective p53 signaling may rely more heavily on glycolytic compensation and thus be more susceptible to glycolysis-targeted strategies. This provides a rationale for considering tumor suppressor status when evaluating metabolic therapeutic approaches in ovarian cancer.

### 5.4 Glycolysis inhibitors as chemosensitizers

The notion that glycolytic reprogramming supports chemoresistance has stimulated interest in glycolysis inhibitors as chemosensitizing agents. Several experimental compounds and pathway-directed interventions have shown promising antitumor effects in ovarian cancer models (112). BAY-876, a selective inhibitor of GLUT1, suppresses glucose uptake, reduces lactate production, and significantly impairs ovarian cancer growth *in vitro* and *in vivo*, indicating that blockade of glucose entry can destabilize the metabolic foundation of tumor survival. Because glucose transport lies upstream of the rest of the pathway, such inhibition may have particularly broad effects on resistant cells that depend on sustained glycolytic substrate supply. Targeting downstream glycolytic amplifiers has also yielded encouraging results. The PFKFB3 inhibitor PFK158 has been reported to promote lipophagy and increase chemosensitivity in gynecologic cancer models, supporting the idea that suppressing glycolytic commitment can weaken adaptive survival programs and improve the efficacy of cytotoxic treatment (69). Similarly, dichloroacetic acid, which functionally opposes PDK-mediated inhibition of pyruvate dehydrogenase, can shift metabolism away from aerobic glycolysis and increase apoptosis in ovarian cancer cells by affecting mitochondrial function (113). These findings suggest that reversing the Warburg-like phenotype may lower the threshold for chemotherapy-induced cell death. Other agents further reinforce the therapeutic promise of metabolic intervention. JQ1, for example, has been shown to suppress ovarian tumor growth through downregulation of LDHA, indicating that interference with lactate-generating output can produce meaningful antitumor effects (71). Taken together, these studies suggest that glycolysis-targeted therapies may function not only as direct growth inhibitors but also as sensitizers that disrupt the metabolic adaptations underlying treatment failure. Although most evidence remains preclinical, the cumulative data support the concept that targeting glycolysis represents a rational strategy to enhance chemotherapy response and potentially overcome resistance in ovarian cancer.

## 6 Immune crosstalk of glycolytic reprogramming in ovarian cancer

Emerging evidence indicates that glycolytic reprogramming not only underpins chemotherapy resistance but also shapes the immune landscape of ovarian cancer (86, 114). Metabolic adaptations that allow tumor cells to survive cytotoxic stress—such as enhanced glucose uptake, lactate accumulation, and redox balance maintenance—simultaneously create a microenvironment conducive to immune suppression (115, 116). Lactate secretion, nutrient competition, and inflammatory signaling can remodel macrophages, induce PD-L1 expression, and impair T-cell effector function. Therefore, chemotherapy resistance and immune evasion are interconnected consequences of the same glycolysis-driven metabolic program, highlighting the need to consider metabolic, therapeutic, and immunological axes together when interpreting tumor progression and designing combination strategies. **Table 2** summarizes representative experimental studies defining the major molecular drivers of glycolytic reprogramming in ovarian cancer, with particular emphasis on GLUT1, HK2, PFKFB3, PDK1, LDHA, and their associated signaling networks.

**Table 2:** Representative studies linking glycolysis to immune crosstalk and immune evasion in ovarian cancer.

Study	Year	Immune component	Glycolysis-related mechanism	Main findings	Implication
Xu et al.	2023	Tumor-associated macrophages (TAMs)/PD-L1 axis	Tumor-derived lactate activates macrophages	Lactate stimulated an IL-1β-centered loop in macrophages, which enhanced tumor cell PD-L1 expression through NF-κB and promoted CCL2-dependent macrophage recruitment	Strong evidence that glycolytic output drives immune escape
Shang et al. (72)	2020	CD4+ T cells/Treg polarization	Ovarian cancer-induced glycolytic rewiring in T cells	Coculture with ovarian cancer cells increased glucose uptake and glycolysis in CD4+ T cells and promoted CD4+CD25+Foxp3+ Treg differentiation; TLR8 activation reversed this effect	Supports tumor-driven metabolic programming of immunosuppressive T cells
Zhao et al. (73)	2025	TAM polarization/anti-PD-L1 response	Glycolysis-related macrophage reprogramming	UBE2I depletion promoted M1 polarization and increased anti-PD-L1 efficacy in ovarian cancer models	Suggests metabolism-based remodeling can improve immunotherapy response
Vidoni et al. (49)	2023	Inflammatory microenvironment	IL-6–glycolysis interaction	IL-6 exacerbated malignant behavior in the setting of glycolysis-linked autophagy suppression; resveratrol attenuated this process	Supports inflammatory-glycolytic coupling in ovarian cancer progression
Xu et al.	2023	Macrophage–tumor crosstalk	Lactate–IL-1β–PD-L1 loop	IL-1β neutralization suppressed tumor growth and synergized with anti-PD-L1 therapy	Provides translational rationale for combining metabolic and immunologic targeting

### 6.1 Glycolysis and the immunosuppressive ovarian tumor microenvironment

Glycolytic reprogramming in ovarian cancer is increasingly recognized as an important determinant of the immune state of the tumor microenvironment. Rather than remaining confined to tumor cell-intrinsic metabolism, enhanced glucose uptake and lactate production reshape local immune conditions through nutrient competition, extracellular acidification, and inflammatory signaling (117–119). As a result, glycolysis can promote a microenvironment that favors immune tolerance over effective antitumor surveillance. In ovarian cancer, this issue is particularly relevant because the disease commonly develops within an ascites-rich and highly immunosuppressive peritoneal milieu, where tumor cells, macrophages, and lymphocytes are in constant metabolic interaction. A key conceptual point is that glycolysis-driven immune remodeling does not necessarily require direct suppression of every immune cell type by the same mechanism. Instead, ovarian cancer appears to use multiple glycolysis-linked outputs, including lactate accumulation, cytokine induction, and altered glucose handling, to generate a broader immunosuppressive ecosystem. Current experimental evidence in ovarian cancer most strongly supports two major axes: a lactate–macrophage–PD-L1 circuit and a tumor cell–CD4+ T-cell metabolic reprogramming circuit (120–123). Together, these studies indicate that glycolysis is not only a fuel pathway for ovarian cancer growth, but also a mediator of immune escape.

### 6.2 Lactate-driven macrophage remodeling and immune escape

One of the clearest examples of glycolysis-linked immune crosstalk in ovarian cancer is the interaction between tumor-derived lactate and tumor-associated macrophages (123–125). Xu and colleagues demonstrated that lactate produced by ovarian cancer cells stimulates macrophages to activate an NLRP3-dependent inflammatory program, leading to IL-1β release. This macrophage-derived IL-1β in turn enhances tumor cell PD-L1 expression through NF-κB, while also promoting CCL2 production and further macrophage recruitment. The result is a self-reinforcing immunosuppressive loop in which glycolytic output from tumor cells drives macrophage remodeling, and macrophages then amplify checkpoint-mediated immune evasion. This mechanism is particularly important because it links a classic metabolic end product, lactate, to a therapeutically relevant immune checkpoint phenotype. In other words, lactate is not merely a byproduct of aerobic glycolysis but an active signal that helps establish an immune-permissive niche for tumor progression. Notably, the same study showed that IL-1β neutralization significantly restrained tumor growth and acted synergistically with anti-PD-L1 therapy in tumor-bearing mouse models, suggesting that interruption of this metabolic-inflammatory loop may enhance immune checkpoint blockade in ovarian cancer (126). These findings provide one of the strongest experimental arguments that glycolytic reprogramming contributes directly to immune escape rather than only indirectly through aggressive growth.

### 6.3 Glycolytic crosstalk with T-cell dysfunction and Treg polarization

In addition to macrophages, ovarian cancer can reshape the metabolic state of CD4+ T cells in ways that favor immune suppression. Shang and colleagues showed that coculture with SKOV3 ovarian cancer cells induced a marked increase in glycolysis-related gene expression in human CD4+ T cells, along with increased glucose uptake and lactate production. This metabolic rewiring was accompanied by enhanced differentiation toward CD4+CD25+Foxp3+ regulatory T cells, indicating that ovarian cancer cells can promote an immunosuppressive T-cell phenotype through metabolic control. This observation is notable for two reasons. First, it shows that glycolysis-related reprogramming in ovarian cancer extends beyond tumor cells themselves and can be imposed on immune cells in the surrounding environment (127). Second, it suggests that the metabolic state of T cells is functionally coupled to their immunologic behavior. In this system, TLR8 activation could reverse aspects of the glycolytic and functional reprogramming of CD4+ T cells, implying that the immunosuppressive phenotype induced by ovarian cancer is at least partly metabolically plastic and potentially reversible (72). Related work further supports a causal relationship between glucose metabolism and Treg suppressive function in the ovarian cancer setting. Beyond macrophages and Tregs, emerging evidence indicates that glycolysis also impacts CD8+ T cells, NK cells, and dendritic cells, broadening the immunosuppressive landscape of ovarian cancer. CD8+ T cells exposed to a glucose-depleted, lactate-rich tumor microenvironment often exhibit impaired effector functions, reduced cytokine production, and increased exhaustion-associated features, limiting cytotoxic activity. Tumor-derived glucose consumption can metabolically restrict T cells and reduce IFN-γ production, while lactate-rich conditions have been associated with impaired CD8+ T-cell infiltration and function (128, 129). Similarly, natural killer (NK) cells can be metabolically suppressed by high lactate and acidic conditions, reducing their cytotoxicity against tumor cells (130, 131). Dendritic cells (DCs) may also experience tumor metabolism-induced stress, impairing antigen processing, antigen presentation, and subsequent T-cell priming (132). Collectively, these findings suggest that aerobic glycolysis in ovarian cancer may orchestrate a broad immunosuppressive network, affecting both innate and adaptive immune components beyond macrophages and Tregs. Although direct ovarian cancer-specific evidence remains limited for CD8+ T cells, NK cells, and DCs, future studies employing single-cell analysis, spatial profiling, immunocompetent models, and metabolic tracing will be crucial to delineate how glycolysis differentially shapes these immune subsets and to identify potential metabolic intervention points for restoring antitumor immunity.

### 6.4 Glycolysis and macrophage polarization

A second macrophage-centered theme is the relationship between glycolytic control and M1/M2 polarization. Recent work by Zhao and colleagues showed that UBE2I depletion reprograms glycolysis in a way that promotes polarization of tumor-associated macrophages toward the M1 phenotype, suppresses tumor progression, and enhances the efficacy of anti-PD-L1 therapy in ovarian cancer models. This study is important because it moves beyond descriptive associations and suggests that manipulating metabolism-linked signaling in the tumor context can actively redirect macrophage identity toward a more antitumor state. More broadly, these findings support the view that macrophage polarization in ovarian cancer is metabolically regulated and therapeutically actionable. While many prior studies established that M2-skewed macrophages correlate with poor outcome in ovarian cancer, the newer glycolysis-focused data indicate that metabolic rewiring is one of the mechanisms through which this polarization state can be established or reversed (133). Thus, glycolysis in ovarian cancer should be considered not only as a tumor-cell growth program but also as a determinant of macrophage functional orientation within the tumor microenvironment.

### 6.5 Inflammatory signaling links glycolysis to immune remodeling

Inflammatory mediators appear to be a major bridge between glycolytic activity and immune dysfunction in ovarian cancer. The IL-1β-centered macrophage loop described above provides one direct example in which a glycolysis-associated metabolite, lactate, induces inflammatory signaling that feeds back to intensify immune escape (134–137). In parallel, ovarian cancer-related studies of CD4+ T cells implicate mTOR-linked glucose metabolism in the maintenance of suppressive lymphocyte function, further highlighting that metabolic pathways and inflammatory or immune signaling are deeply intertwined. Although ovarian cancer-specific experimental evidence is still less extensive than in some other tumor types, current studies strongly suggest that glycolysis-related immune remodeling is not mediated by lactate alone. Instead, it likely involves a broader network of inflammatory effectors, including NF-κB, IL-1β, and other cytokine programs that shape macrophage recruitment, checkpoint expression, and T-cell behavior. This view is consistent with the emerging notion that immunometabolic adaptation in ovarian cancer arises from reciprocal communication among tumor cells and immune cells, rather than from a single linear pathway.

### 6.6 Current limitations in understanding glycolysis–immune interactions in ovarian cancer

Despite these advances, the current literature on glycolysis–immune crosstalk in ovarian cancer remains relatively limited. Most mechanistic studies focus on a small number of immune cell populations, especially macrophages and CD4+ T cells, and many experiments rely on coculture systems or selected preclinical models. Direct evidence covering other important immune components in ovarian cancer, such as CD8+ T cells, NK cells, dendritic cells, and myeloid-derived suppressor cells, is still sparse in the context of glycolytic reprogramming.

Another limitation is that the field still lacks sufficient single-cell, spatial, and immunocompetent *in vivo* analyses capable of defining how glycolytic gradients, lactate distribution, and nutrient competition shape immune heterogeneity across the ovarian tumor ecosystem. As a result, the current picture is mechanistically suggestive but still incomplete. Future studies integrating metabolic tracing, spatial multi-omics, and immune-intact models will be essential for clarifying how glycolysis drives immune evasion across different ovarian cancer subtypes and treatment settings.

## 7 Therapeutic implications of targeting glycolysis in ovarian cancer

**Table 3** summarizes current preclinical strategies targeting glycolysis in ovarian cancer and outlines their potential therapeutic relevance, either as direct antitumor approaches or as combination partners for chemotherapy and immunotherapy.

**Table 3:** Potential therapeutic strategies targeting glycolysis in ovarian cancer.

Agent/strategy	Target	Experimental setting	Main antitumor effects	Potential translational value
BAY-876	GLUT1	Ovarian cancer cell lines, xenografts, PDXs	Reduced glucose uptake, lactate production, anchorage-independent growth, and tumorigenicity	Promising approach for tumors with strong glucose transport dependency
PFK158	PFKFB3	Gynecologic cancer preclinical models	Suppressed glycolysis, promoted lipophagy, increased chemosensitivity	Suitable for combination with chemotherapy
Dichloroacetic acid (DCA)	PDK axis/pyruvate dehydrogenase reactivation	Ovarian cancer cells	Shifted metabolism away from glycolysis, improved mitochondrial function, increased apoptosis	May reverse Warburg-like metabolism and sensitize tumors to therapy
JQ1	LDHA-associated glycolytic output	Ovarian cancer cells and tumor models	Suppressed tumor growth and downregulated LDHA	Supports targeting lactate-producing metabolic output
HK2 targeting	HK2	Cell and xenograft studies	Reduced proliferation, migration, invasion, and stemness	Attractive because HK2 links metabolism with malignant phenotype
LDHA suppression via miR-383 axis	LDHA	Cell studies	Reduced aerobic glycolysis, proliferation, and invasion	Supports LDHA as a metabolic and functional target
PFKFB3 suppression via miR-206 axis	PFKFB3	Cell studies	Reduced proliferation and migration	Suggests ncRNA-guided targeting may complement direct inhibitors
Metabolic targeting + anti-PD-L1	Lactate/TAM/IL-1β/PD-L1 axis	Preclinical immune studies	Reduced immune suppression and enhanced checkpoint blockade response	Strong rationale for immunometabolic combination strategies

### 7.1 Direct inhibition of glycolytic enzymes and transporters

The mechanistic and functional evidence summarized above strongly supports glycolysis as a therapeutic vulnerability in ovarian cancer. Because ovarian cancer cells frequently rely on enhanced glucose uptake, sustained glycolytic commitment, altered pyruvate fate, and high lactate output, several nodes within this pathway have emerged as candidate intervention points. These include GLUT1, HK2, PFKFB3, PDK1, and LDHA, each of which occupies a distinct position in glycolytic control and therefore offers different translational advantages. Broadly, inhibition of upstream substrate entry may deprive tumor cells of metabolic input, whereas inhibition of downstream amplifiers or metabolic endpoints may interfere with specific adaptive outputs such as lactate accumulation, redox maintenance, and survival signaling.

Among currently studied strategies, GLUT1 inhibition is supported by some of the strongest preclinical evidence. The selective GLUT1 inhibitor BAY-876 suppresses basal and stress-regulated glycolysis, reduces lactate production, and markedly impairs ovarian cancer growth *in vitro* as well as tumorigenicity in xenograft and patient-derived xenograft models. These findings indicate that disruption of glucose entry alone can destabilize the metabolic foundation of ovarian cancer growth, especially in tumors with pronounced glycolytic dependency. Because GLUT1 acts at the entry point of the pathway, its inhibition may also blunt multiple downstream adaptive programs simultaneously. Downstream glycolytic regulators are also attractive. PFKFB3 is particularly compelling because it amplifies glycolytic commitment at a major regulatory checkpoint, and the inhibitor PFK158 has shown activity in gynecologic cancer models by suppressing glycolysis, promoting lipophagy, and enhancing treatment response. Likewise, targeting PDK1 offers a way to remodel pyruvate fate and counter the Warburg-like phenotype by restoring mitochondrial oxidation, while LDHA inhibition may reduce lactate-generating output and its downstream protumor consequences. Although direct clinical translation remains limited, these studies collectively support the concept that ovarian cancer glycolysis can be therapeutically targeted at multiple levels rather than through a single universal metabolic drug.

### 7.2 Combination strategies with chemotherapy

One of the most promising translational implications of glycolysis targeting is its potential to enhance chemosensitivity. Because glycolytic reprogramming helps ovarian cancer cells maintain ATP production, buffer oxidative stress, and survive treatment-induced injury, blocking this pathway may lower the threshold for chemotherapy-mediated cell death. This rationale is particularly relevant in ovarian cancer, where platinum resistance remains a major cause of relapse and treatment failure. Preclinical data support this strategy. Inhibition of glycolytic commitment through PFKFB3 targeting has been shown to increase chemosensitivity in gynecologic cancer models, suggesting that metabolic disruption can weaken adaptive survival programs that normally protect tumor cells during therapy. Similarly, dichloroacetic acid (DCA), which functionally opposes PDK-driven suppression of pyruvate dehydrogenase, promotes apoptosis in ovarian cancer cells by regulating mitochondrial function and appears especially relevant in the context of tumors with distinct metabolic phenotypes (138, 139). These findings imply that shifting cells away from glycolysis and toward oxidative metabolism can make them less able to tolerate cytotoxic stress. Other agents further reinforce the principle that metabolic intervention can sensitize ovarian cancer to treatment. JQ1 suppresses ovarian tumor growth at least in part through downregulating LDHA, reducing lactate production, and decreasing cellular energy supply (140). While such agents may not have been developed primarily as glycolysis inhibitors, their antitumor effects illustrate how disrupting metabolic endpoints can compromise tumor resilience. Taken together, these studies suggest that combining chemotherapy with glycolysis-targeted agents may be a rational means of overcoming metabolic resistance and improving treatment efficacy in ovarian cancer.

### 7.3 Combination strategies with immunotherapy

The emerging connection between glycolysis and immune evasion also raises the possibility that metabolic intervention could improve immunotherapy response in ovarian cancer. This concept is especially appealing because current immune checkpoint blockade strategies have shown limited efficacy in unselected ovarian cancer populations, suggesting that the immunosuppressive tumor microenvironment remains a major obstacle. By reducing lactate production, altering macrophage polarization, and weakening checkpoint-promoting inflammatory loops, glycolysis-targeted approaches may help convert a metabolically hostile immune niche into one more permissive for antitumor immunity. Experimental evidence supports this logic. Xu and colleagues showed that tumor-derived lactate drives a macrophage–IL-1β–PD-L1 loop in ovarian cancer, and that IL-1β neutralization not only curbed tumor growth but also synergized with anti-PD-L1 therapy *in vivo*. More recently, UBE2I depletion was reported to promote M1 macrophage polarization through glycolysis-related reprogramming and to enhance the efficacy of anti-PD-L1 immunotherapy in ovarian cancer models (73). These findings provide proof-of-concept that interventions disrupting glycolysis-linked immune remodeling can potentiate checkpoint blockade, even if the optimal clinical strategy has yet to be defined. From a translational perspective, this suggests that glycolysis-targeted therapy may serve not only as a direct antitumor approach but also as an immunometabolic adjuvant. Future combination regimens might include checkpoint blockade plus agents that reduce glucose uptake, limit lactate generation, or reprogram macrophage metabolism. Such strategies could be especially valuable in tumors characterized by high lactate burden, strong PD-L1 induction, or prominent macrophage-driven immune suppression.

### 7.4 Challenges in clinical translation

Despite the strong preclinical rationale, several challenges currently limit the clinical translation of glycolysis-targeted therapy in ovarian cancer. First, glycolysis is a fundamental metabolic pathway in normal tissues, raising concerns about on-target toxicity and therapeutic selectivity. While tumor cells may display heightened dependence on glycolysis, especially under stress, normal proliferative tissues and immune cells also require glucose metabolism, which complicates dosing and combination design. Second, ovarian cancer is metabolically heterogeneous. Not all tumors rely on glycolysis to the same extent, and many retain substantial metabolic plasticity, allowing compensation through oxidative phosphorylation, lipid metabolism, or other adaptive routes when glycolysis is inhibited. This means that single-agent glycolysis inhibition may be insufficient in some contexts and that effective treatment will likely require biomarker-guided patient stratification. Markers such as GLUT1, HK2, PFKFB3, LDHA, lactate-related signatures, or immunometabolic phenotypes may eventually help identify the subsets most likely to benefit. Third, most available evidence remains preclinical. The field still lacks robust clinical trials specifically designed around glycolysis dependency in ovarian cancer, and the best way to combine metabolic inhibitors with chemotherapy or immunotherapy remains unresolved. Future progress will depend on better pharmacologic tools, careful toxicity evaluation, mechanistically informed combination strategies, and integration of metabolic profiling into trial design. Even so, the current body of work strongly suggests that targeting glycolysis represents a promising translational avenue, particularly when deployed as part of rational combination therapy rather than as an isolated intervention. Although much of the current evidence is derived from cellular and preclinical models, several clinical studies have reported associations between glycolytic markers and patient outcomes in ovarian cancer. Elevated GLUT1 expression has been correlated with advanced tumor stage, high-grade serous histology, and shorter overall survival (23, 141). Similarly, high HK2 or LDHA levels have been associated with poor prognosis, chemoresistance, and reduced progression-free survival (30, 142, 143). These findings support the translational relevance of glycolytic reprogramming and underscore the potential utility of GLUT1, HK2, and LDHA as prognostic and predictive biomarkers in patient stratification. Future studies integrating clinical cohorts, longitudinal monitoring, and spatial profiling will be essential to validate these markers for precision therapy.

## 8 Challenges and future perspectives

### 8.1 Need for subtype-specific metabolic profiling

One of the major challenges in this field is the metabolic heterogeneity of ovarian cancer. Ovarian cancer is not a single biologic entity but a group of diseases with distinct histologic, molecular, and clinical features, and it is unlikely that all subtypes depend on glycolysis to the same extent. Even within high-grade serous ovarian cancer, substantial intertumoral and intratumoral variability exists in nutrient use, mitochondrial activity, and stress adaptation. This heterogeneity may help explain why some tumors appear highly sensitive to glycolysis inhibition whereas others are more capable of metabolic compensation. Future studies should therefore move beyond generalized descriptions of the Warburg effect and define glycolytic dependency in a subtype-specific and context-dependent manner. Such efforts will be essential for identifying which ovarian cancer populations are most likely to benefit from metabolic targeting. A related issue is that metabolic state is not fixed over time. Ovarian cancer cells can dynamically shift between glycolysis-dominant and more oxidative programs in response to detachment, ascites exposure, hypoxia, stromal input, and treatment pressure. Accordingly, future work should not only compare baseline metabolic phenotypes across subtypes, but also determine how these phenotypes evolve during progression, recurrence, and therapy resistance. This temporal dimension of metabolic plasticity is likely to have major implications for biomarker development and treatment timing.

### 8.2 Need for multi-omics and single-cell resolution

A second major priority is the application of single-cell and multi-omics approaches to resolve the complexity of glycolytic reprogramming in ovarian cancer. Most current studies infer metabolic behavior from bulk gene expression, selected protein markers, or functional assays performed in established cell lines. While these studies have been invaluable, they cannot fully capture the spatial and cellular heterogeneity of the ovarian tumor ecosystem (144–146). Tumor cells, macrophages, fibroblasts, mesothelial cells, and lymphocytes likely experience different nutrient availabilities and metabolic pressures even within the same lesion, and these localized differences may profoundly shape progression and therapy response. Future investigations should integrate single-cell transcriptomics, spatial transcriptomics, metabolomics, proteomics, and metabolic tracing to map glycolytic states with much greater precision. This will be particularly important for clarifying how lactate gradients, glucose competition, and pyruvate utilization vary across tumor nests, ascitic environments, and metastatic implants. Such approaches may also reveal rare but clinically important subpopulations, such as highly glycolytic stem-like cells or immunosuppressive macrophage subsets that are disproportionately responsible for recurrence and immune escape. In this regard, the next phase of research should aim not merely to confirm that glycolysis is upregulated in ovarian cancer, but to define exactly where, when, and in which cells it matters most.

### 8.3 Biomarker development for patient stratification

For glycolysis-targeted therapy to become clinically useful, there is a pressing need for robust biomarkers that can identify tumors with meaningful metabolic dependency. At present, most studies rely on individual markers such as GLUT1, HK2, PFKFB3, PDK1, or LDHA, but it is unlikely that any single molecule will be sufficient to capture the full complexity of glycolytic reliance. More informative biomarkers may need to combine gene or protein expression with functional indicators such as lactate production, imaging-based glucose uptake, or immunometabolic signatures linked to macrophage remodeling and checkpoint activation. Patient stratification may also need to incorporate molecular context. For example, tumors with dysregulated p53, activated PI3K/AKT/HIF-1α, or strong PFKFB3 signaling may display distinct patterns of glycolytic reliance and treatment response. Similarly, tumors with evidence of lactate-driven immune suppression or Treg-favoring metabolic crosstalk may be particularly suitable for combined metabolic-immunotherapy approaches (147–149). Developing such biomarker frameworks will be essential if glycolysis-targeted interventions are to move beyond broad preclinical promise and toward precision oncology.

### 8.4 Rational design of metabolism-immunotherapy combinations

An especially important future direction is the rational integration of glycolysis-targeted strategies with immunotherapy. Current evidence suggests that glycolytic reprogramming contributes to immune evasion through lactate accumulation, macrophage remodeling, PD-L1 induction, and T-cell metabolic dysfunction (150–152). These findings imply that metabolic intervention could improve immune checkpoint blockade not only by weakening tumor growth, but also by restoring a more permissive immune microenvironment. However, the optimal way to implement this concept remains unclear. Several questions require attention. It will be important to determine which metabolic nodes are most effective for immune remodeling, whether treatment should focus on tumor cells, immune cells, or both, and how timing should be coordinated with checkpoint inhibitors or chemotherapy. For example, reducing tumor-derived lactate may alleviate macrophage-driven PD-L1 induction, whereas reprogramming immune-cell glucose metabolism may be more relevant for improving T-cell function. Future combination strategies should therefore be designed on the basis of mechanism rather than empirical drug stacking. The goal should be to build immunometabolic regimens that simultaneously target tumor fitness and immune suppression.

### 8.5 From descriptive association to mechanistic and translational validation

Although the literature on glycolysis in ovarian cancer has grown substantially, much of it still remains descriptive or based on limited model systems. Many studies identify correlations between glycolytic enzymes and aggressive phenotypes, but fewer establish complete mechanistic chains connecting metabolic rewiring to clinically relevant outcomes across multiple experimental levels. This is particularly true for immune crosstalk, where the number of ovarian cancer-specific mechanistic studies remains relatively small. To advance the field, future work will need to move from association toward mechanistic closure and translational validation. This will require more sophisticated model systems, including patient-derived organoids, immunocompetent mouse models, longitudinal sampling, and clinically annotated cohorts. It will also require intervention studies that test whether targeting glycolysis can meaningfully alter progression, recurrence, or therapeutic response in settings that better approximate human disease. Ultimately, the value of this field will depend not only on demonstrating that glycolytic reprogramming is common in ovarian cancer, but on proving that it can be exploited to improve patient outcomes in a reproducible and biologically informed manner.

## 9 Conclusion

Glycolytic reprogramming represents a central adaptive program in ovarian cancer, extending beyond elevated glucose consumption to orchestrate tumor metabolism, chemoresistance, and immune modulation. By coordinating key metabolic nodes—GLUT1, HK2, PFKFB3, PDK1, and LDHA—through oncogenic, microenvironmental, and ncRNA-mediated pathways, ovarian cancer cells acquire enhanced proliferation, invasive dissemination, stem-like plasticity, and survival under therapeutic stress. Importantly, this metabolic shift simultaneously reshapes the immune microenvironment, promoting lactate-driven macrophage remodeling, PD-L1-mediated immune escape, and T-cell metabolic suppression. Compared with previous reviews, this work integrates these immunometabolic circuits and emphasizes their relevance to clinical translation, highlighting potential vulnerabilities for combined glycolysis-targeted, chemotherapy, and immunotherapy strategies.

Key challenges remain, including metabolic heterogeneity, compensatory pathways, and limited biomarker frameworks, underscoring the need for subtype-specific profiling, single-cell and spatial multi-omics, and mechanism-informed therapeutic design. Moving forward, future studies should focus on validating glycolysis–immune crosstalk using immunocompetent mouse models, organoids, and PDX models; developing combined GLUT1/HK2/PFKFB3/LDHA/lactate-related biomarkers for patient stratification; integrating lactate imaging, metabolomics, and single-cell/spatial transcriptomics; and testing rational combinations of glycolysis inhibitors with platinum chemotherapy, PARP inhibitors, anti-angiogenic therapy, or immune checkpoint blockade.

Overall, understanding glycolysis as a hub connecting metabolism, tumor aggressiveness, and immune evasion provides a framework for rationally translating mechanistic insights into clinical interventions and precision treatment strategies in ovarian cancer.
